# Sirtuin 1 ameliorates defenestration in hepatic sinusoidal endothelial cells during liver fibrosis via inhibiting stress‐induced premature senescence

**DOI:** 10.1111/cpr.12991

**Published:** 2021-02-01

**Authors:** Xiaoying Luo, Yangqiu Bai, Shuli He, Suofeng Sun, Xiaoke Jiang, Zhiyu Yang, Di Lu, Peiru Wei, Yuan Liang, Cong Peng, Yaru Wang, Ruli Sheng, Shuangyin Han, Xiuling Li, Bingyong Zhang

**Affiliations:** ^1^ Department of Gastroenterology Henan Provincial People's Hospital People's Hospital of Zhengzhou University School of Clinical Medicine Henan University Zhengzhou China; ^2^ Microbiome Laboratory Henan Provincial People's Hospital People's Hospital of Zhengzhou University Zhengzhou China

**Keywords:** defenestration, hepatic sinusoidal endothelial cell, premature senescence, progerin, sirtuin 1

## Abstract

**Objective:**

Premature senescence is related to progerin and involves in endothelial dysfunction and liver diseases. Activating sirtuin 1 (SIRT1) ameliorates liver fibrosis. However, the mechanisms of premature senescence in defenestration of hepatic sinusoidal endothelial cells (HSECs) and how SIRT1 affects HSECs fenestrae remain elusive.

**Methods:**

We employed the CCl_4_‐induced liver fibrogenesis rat models and cultured primary HSECs in vitro, administered with the SIRT1‐adenovirus vector, the activator of SIRT1 and knockdown NOX2. We measured the activity of senescence‐associated β‐galactosidase (SA‐β‐gal) in HSECs. Meanwhile, the protein expression of SIRT1, NOX2, progerin, Lamin A/C, Ac p53 K381 and total p53 was detected by Western blot, co‐immunoprecipitation and immunofluorescence.

**Results:**

In vivo, premature senescence was triggered by oxidative stress during CCl_4_‐induced HSECs defenestration and liver fibrogenesis, whereas overexpressing SIRT1 with adenovirus vector lessened premature senescence to relieve CCl_4_‐induced HSECs defenestration and liver fibrosis. In vitro, HSECs fenestrae disappeared, with emerging progerin‐associated premature senescence; these effects were aggravated by H_2_O_2_. Nevertheless, knockdown of NOX2, activation of SIRT1 with resveratrol and SIRT1‐adenovirus vector inhibited progerin‐associated premature senescence to maintain fenestrae through deacetylating p53. Furthermore, more Ac p53 K381 and progerin co‐localized with the abnormal accumulation of actin filament (F‐actin) in the nuclear envelope of H_2_O_2_‐treated HSECs; in contrast, these effects were rescued by overexpressing SIRT1.

**Conclusion:**

SIRT1‐mediated deacetylation maintains HSECs fenestrae and attenuates liver fibrogenesis through inhibiting oxidative stress‐induced premature senescence.

## Graphical abstract

SIRT1‐mediated deacetylation of p53 relieves NOX2‐dependent oxidative stress and inhibits progerin‐associated premature senescence to maintain fenestrae in HSECs.

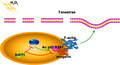

## 1 INTRODUCTION

Premature senescence involves in cellular dysfunction and various chronic diseases, and its character is the inhibition of cell proliferation in advance when suffering from noxious stimuli.^1^ Emerging evidence confirms that due to old age, the phenotypes of all hepatic cells have changed, such as the loss of fenestration in hepatic sinusoidal endothelial cells (HSECs). Furthermore, the defenestration and capillarization of HSECs are observed in some premature senescence‐related disease paradigms.^2^, ^3^ The research indicates that premature senescence in HSECs may be closely related to HSECs defenestration and liver fibrogenesis. Hence, elucidation of the underlying mechanisms for premature senescence may be a key to our understanding of defenestration in HSECs and liver fibrosis pathogenesis.

The contraction and dilatation of fenestrae in HSECs are regulated by actin cytoskeleton (including F‐actin).^4^ Our previous studies reveal that oxidative damage facilitates HSECs defenestration during liver fibrogenesis via F‐actin remodelling.^5^, ^6^ Novel findings show that lamins and their associated proteins, which regulate nucleoskeleton and cytoskeleton, affect cellular differentiation and senescence.^7^, ^8^ Especially, progerin is a mutant Lamin A protein, and the accumulation of progerin brings about abnormal nucleoskeleton and cellular premature senescence, so as to promote the occurrence and development of chronic liver diseases.^7^, ^8^, ^9^ Thus, we speculate that progerin may contribute to premature senescence‐associated HSECs defenestration via abnormal cytoskeleton remodelling.

Sirtuin 1 (SIRT1) is an essential protector against oxidative stress and senescence to reverse the progression of chronic liver diseases.^10^ Recent studies emphasize that overexpressing or activating SIRT1 inhibits hepatic senescence and activation of HSCs to ameliorate liver fibrosis.^11^, ^12^ Besides, a significant finding demonstrates that the activation of SIRT1 prevents the endothelial cells from oxidative stress‐induced senescence and dysfunction.^13^, ^14^ Nevertheless, the effects of SIRT1 on premature senescence and defenestration in HSECs during liver fibrogenesis remain elusive.

Herein, our present study investigates the underlying mechanisms and the intervening target linking premature senescence and HSECs defenestration, and the role of SIRT1 in HSECs defenestration in vitro and in vivo. We specifically focus on the SIRT1‐mediated deacetylation, which may influence premature senescence‐associated defenestration of HSECs in liver fibrogenesis.

## 2 MATERIALS AND METHODS

### 2.1 Reagents and antibodies

The reagents included carbon tetrachloride (Sigma‐Aldrich, 56‐23‐5), 30% H_2_O_2_ (Hydrogen peroxide 30%, Sigma‐Aldrich, 1.07298), selisistat (EX‐527, MedChemExpress, 49843‐98‐3), resveratrol (SRT501, MedChemExpress, 501‐36‐0), DAPI (Sigma‐Aldrich, D9542), Alexa Fluor™ 647 Phalloidin (Thermo, A22287), protease cocktails inhibitor (Beyotime, P1005) and PMSF (Phenylmethanesulphonyl fluoride, Beyotime, ST506).

The primary antibodies included anti‐α‐SMA (Boster, BM0002), anti‐vWF (Santa Cruz, SC‐365712), anti‐CD32b (ZEN‐bioscience, 382560), anti‐CD31 (PECAM‐1, Santa Cruz, sc‐18916), anti‐CD31 (Abcam, ab33858), anti‐NOX2 (Proteintech, 19013‐1‐AP), anti‐NOX4 (Proteintech, 14347‐1‐AP), anti‐Lamin A/C (Cell Signaling Technology, 4777S), anti‐Lamin B1 (Proteintech, 66095‐1‐Ig), anti‐progerin (Santa Cruz, sc‐81611), anti‐p53 (Abcam, ab131442), anti‐p53 (acetyl K381; Abcam, ab61241), anti‐SIRT1 (Abcam, ab110304), anti‐Histone H3 (Proteintech, 17168‐1‐AP) and anti‐GAPDH (Proteintech, 60004‐1). HRP‐conjugated Affinipure Goat Anti‐Mouse IgG (H + L; Proteintech, SA00001‐1), HRP‐conjugated Affinipure Goat Anti‐Rabbit IgG (H + L; Proteintech, SA00001‐2), FITC‐labelled goat anti‐rabbit IgG (H + L; Beyotime, a0562) and Cy3‐labelled goat anti‐mouse IgG (H + L; Beyotime, a0521) were used for secondary antibodies.

### 2.2 Animal experimental design

The animal experiments were approved by the Committee on the Ethics of Animal Experiments of Southern Medical University (the ethical approval code: 1912033). Sprague‐Dawley (SD) rats were provided by the Laboratory Animal Center (Henan University of Chinese Medicine, China), and its certificate number was 41003100006844. Rats were housed under a 12:12 h light/dark cycle at 22‐24°C.

#### 2.2.1 Establishment of CCl_4_‐induced liver fibrogenesis rat models

Normal male SD rats (180‐220 g, 6 weeks old) were subjected to intraperitoneal injection of 40% carbon tetrachloride (CCl_4_)‐olive oil solution at 2 ml/kg body weight, twice a week for 28 days. On Day 0, 3, 6 and 28, CCl_4_‐induced rat models were randomly sacrificed (n = 6 per group).

#### 2.2.2 The treatment of SIRT1 adenovirus vector

To investigate the role of SIRT1 in the fenestrae of primary HSECs and liver fibrogenesis, the GFP‐SIRT1‐adenovirus vector and the GFP‐blank vector were produced by Hanbio AdenoVector Institute (Shanghai, China), and the dose of 10^11^ viral particles was injected through a caudal vein to rats 1 week before the intraperitoneal injection of CCl_4_‐olive oil solution. We employed the CCl_4_‐induced liver fibrosis rat models (n = 6 per group for 6 days and n = 6 per group for 28 days). The vehicle group (n = 6 per group for 6 days and n = 6 per group for 28 days) was subjected to intraperitoneal injection of the same volume of olive oil, twice a week for 28 days. The AV‐CTR + CCl_4_ group and the AV‐SIRT1 + CCl_4_ group (n = 6 per group for 6 days and n = 6 per group for 28 days) were subjected to intraperitoneal injection of CCl_4_‐olive oil solution twice a week after administering vectors. On Day 6 and 28, the rat models were randomly sacrificed. The SIRT1 sequences were used: sense (5‐ CGGGCCCTCTAGACTCGAGCGGCCGCATGATTGGCACCGATCCTC‐3).

### 2.3 Histological analysis and immunohistochemistry

Paraffin sections (4 μm) of liver tissue of the model rats were prepared with haematoxylin and eosin (H&E) staining. The rat liver histological inflammation and fibrosis stage are assessed with the Ishak inflammation and fibrosis score (from the supplementary method). Immunohistochemical detection of α‐SMA, vWF and Ac p53 K381 was performed on paraffin sections (3 μm) of liver tissue, and subsequent sections were exposed to HRP‐antibody coloured with DAB, and visualized by microscopy (BX51, Olympus, Japan). The degree of liver fibrosis and the number of α‐SMA‐, vWF‐ or Ac p53 K381‐positive cells were quantified with Image J software.

### 2.4 Immunofluorescence staining

Paraffin sections (3 μm) of liver tissue of the model rats were prepared for immunofluorescence, incubated with primary antibody overnight, followed by the secondary antibody, and then mounted with DAPI. The primary antibodies included anti‐progerin (1:50), anti‐SIRT1 (1:200) and anti‐vWF (1:200). The secondary antibodies included FITC‐labelled goat anti‐rabbit IgG (H + L; 1:200) and Cy3‐labelled goat anti‐mouse IgG (H + L; 1:200).

### 2.5 Cell isolation, identification, culture and treatment

Primary HSECs were isolated from normal male SD rats and identified by SEM, based on modified method.^5^, ^6^ Primary HSECs were cultured in plates with a medium comprising 80% MCDB131 (Gibco, 10372019) and 20% foetal bovine serum (FBS, Biological Industries, 04‐007‐1A). Primary HSECs were stimulated by H_2_O_2_ with a concentration gradient of 0, 1.25, 2.5, 5, 10 μM for 24 hours, or a time gradient of 0, 12, 24, 36, 48 hours.

### 2.6 Measurement of the activity of senescence‐associated β‐galactosidase (SA‐β‐Gal)

The activity of SA‐β‐Gal in primary HSECs was determined using 5‐bromo‐4‐chloro‐3‐indolyl P3‐D‐galactoside (X‐gal), according to the manufacturer instruction (Senescence‐associated β‐galactosidase Staining Kit, Beyotime, C0602). SA‐β‐Gal‐positive cells (blue colour) were counted under a microscope.

### 2.7 Scanning electron microscopy (SEM)

The liver tissue of the model rats and primary HSECs was fixed with 2.5% glutaraldehyde and subsequently dehydrated, and then coated with gold using the coating apparatus, based on the modified method.^5^ Eventually, fenestrae in primary HSECs of samples were observed with SEM at 15‐kV acceleration voltage.

### 2.8 SIRT1 adenovirus transfection

The recombinant adenovirus was produced by Hanbio AdenoVector Institute (Shanghai, China). To construct Flag protein‐tagged SIRT1, full‐length SIRT1 cDNA was amplified from a human cDNA library and fused at its C‐terminus with sequences encoding the monomeric protein. Briefly, the amplified SIRT1 fragment was inserted into the adenoviral vector, which contains the mouse cytomegalovirus (CMV) promoter, using the AdMax system. The resultant Flag‐SIRT1 protein gene fusion was validated by nucleotide sequencing. Flag protein was detected by Western blotting. Primary HSECs were transfected with this adenovirus vector to overexpress SIRT1, according to the manufacturer's instructions. The SIRT1 sequences were used: sense (5‐ CGGGCCCTCTAGACTCGAGCGGCCGCATGATTGGCACCGATCCTC‐3).

### 2.9 Small interfering RNA (siRNA) transfection assay

Primary HSECs were transfected with siRNA to silence NOX2, p53 and progerin, according to the manufacturer instructions. The transfection efficiency was 75%. The following NOX2 siRNA sequences were used: sense (5‐ CCTCCTATGACTTGGAAAT‐3). The following p53 siRNA sequences were used: sense (5‐GGCTCCGACTATACCACTA‐3). The following progerin siRNA sequences were used: sense (5‐GCTCAGTGACTGTGGTTGA‐3).

### 2.10 Extraction of nuclear and cytoplasmic protein of primary HSECs

Nuclear and Cytoplasmic Protein Extraction Kit (Beyotime, P0028) was used to extract nuclear and cytoplasmic protein of primary HSECs (10^7^ cells per group). Nuclear and cytoplasmic protein was processed and detected for Western blotting.

### 2.11 Immunocytochemistry

Paraformaldehyde‐fixed primary HSECs were incubated with primary antibodies, followed by the secondary antibodies, and subsequently mounted with DAPI. The primary antibodies included anti‐NOX2 (1:200), anti‐progerin (1:50) and anti‐Ac p53 K381 (1:200). After incubation with primary antibodies and the secondary antibodies, HSECs were stained with the phallotoxin to detect F‐actin. The number of positive cells was observed by fluorescence microscopy and quantified by Image J software.

### 2.12 Co‐immunoprecipitation (Co‐IP)

Primary HSECs were transfected with SIRT1 adenovirus vectors and were subsequently stimulated with H_2_O_2_ for two days. IP and immunoblotting (IB) were performed as previously described.^5^ The antibodies for IP included anti‐progerin and non‐specific IgG; the antibodies for IB included anti‐progerin, anti‐Ac p53 K381 and anti‐p53.

### 2.13 Western blotting

Primary HSECs were isolated from normal rats and were treated with various stimulators, or were isolated from the model rats. HSECs were lysed in lysis buffer containing protease cocktails inhibitor and PMSF, and centrifuged at 12 000*g*, 4℃, for 15 min. The protein levels of HSECs were detected by Western blotting. The primary antibodies included anti‐vWF (1:1000), anti‐NOX2 (1:1000), anti‐NOX4 (1:1000), anti‐Ac p53 K381 (1:1000), anti‐p53 (1:1000), anti‐progerin (1:25), anti‐Lamin A/C (1:1000), anti‐Lamin B1 (1:1000), anti‐SIRT1 (1:1000), anti‐Histone H3 (1:1000) and anti‐GAPDH (1:1000). The secondary antibodies were HRP‐conjugated Affinipure Goat Anti‐Mouse IgG (H + L; 1:10 000, Proteintech, SA00001‐1) and HRP‐conjugated Affinipure Goat Anti‐Rabbit IgG (H + L; 1:10 000, Proteintech, SA00001‐2). The protein bands were visualized using the Pierce™ ECL Western Blotting Substrate.

### 2.14 Statistical analysis

The data were reported as the mean ± standard deviation (SD) and were analysed by SPSS17.0 software. In the statistical analysis of two groups, a two‐tailed Student's *t* test was utilized, whereas, in the statistical analysis of more than two groups, one‐way ANOVA was performed. *P* < .05 was considered significant.

## 3 RESULTS

### 3.1 Premature senescence is induced by oxidative damage, with the decrease of SIRT1 during defenestration in HSECs of CCl_4_‐induced liver fibrogenesis

**FIGURE 1:**
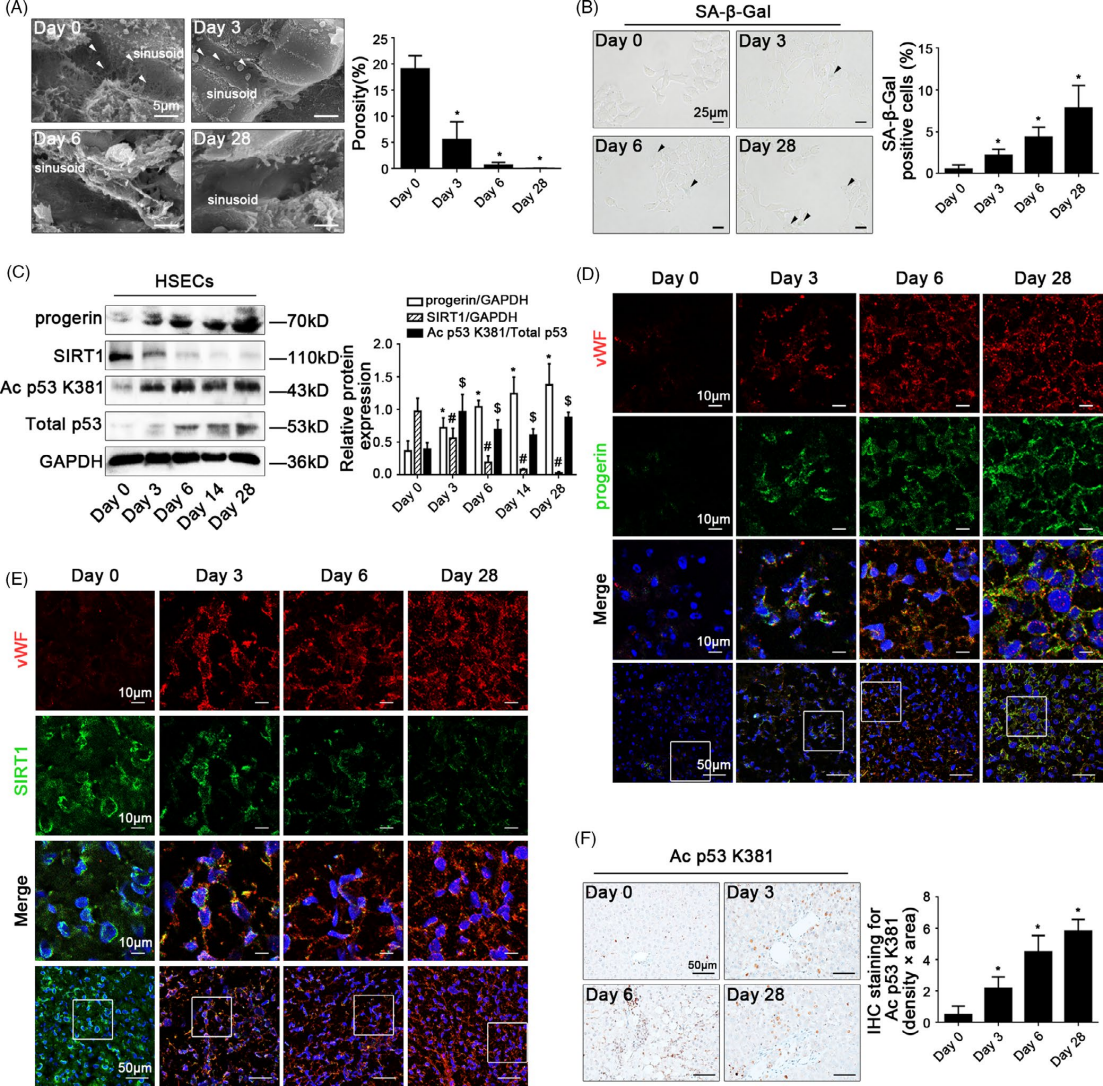
CCl_4_ induces progerin‐associated premature senescence in defenestrated HSECs during liver fibrogenesis. (A) Magnification of scanning electron micrograph (SEM) of hepatic sinusoidal endothelium in CCl_4_‐induced rat models (Day 0, Day 3, Day 6 and Day 28), revealing fenestrae structures in hepatic sinusoidal endothelium (Scale bar: 5 μm). The white triangles indicated fenestrae in hepatic sinusoidal endothelium. The porosity of hepatic sinusoidal endothelium was quantified in the graph, right. ^*^ *P* < .05 vs Day 0. (B) The SA‐β‐Gal activity on primary HSECs, isolated from CCl_4_‐induced rat models (Day 0, Day 3, Day 6 and Day 28) was observed by SA‐β‐Gal staining (Scale bar: 25 μm). The black triangles indicated the SA‐β‐Gal‐positive cells. The SA‐β‐Gal‐positive cells were quantified in the graph, right. ^*^ *P* < .05 vs Day 0. (C) Representative immunoblots of progerin, SIRT1, Ac p53 K381 and total p53 of primary HSECs, isolated from CCl_4_‐induced rat models (Day 0, Day 3, Day 6, Day 14 and Day 28). The relative protein expression of progerin and SIRT1, as well as the ratio of Ac p53 K381 and total p53 protein levels were quantified in the graph, right. ^*^ *P* < .05 vs progerin relative protein level on Day 0; ^#^ *P* < .05 vs SIRT1 relative protein level on Day 0; ^$^ *P* < .05 vs the ratio of Ac p53 K381 and total p53 protein levels on Day 0. (D) The immunofluorescent co‐localization of vWF (red) with progerin (green) of liver biopsy specimens in CCl_4_‐induced rat models (Day 0, Day 3, Day 6 and Day 28), visualized by confocal microscopy (Scale bar: 10 μm, 50 μm). Nuclear was showed by DAPI (blue). (E) The immunofluorescent co‐localization of vWF (red) with SIRT1 (green) of liver biopsy specimens in CCl_4_‐induced rat models (Day 0, Day 3, Day 6 and Day 28), visualized by confocal microscopy (Scale bar: 10 μm, 50 μm). Nuclear was showed by DAPI (blue). (F) The immunohistochemical (IHC) staining for Ac p53 K381 of liver biopsy specimens in CCl_4_‐induced rat models (Day 0, Day 3, Day 6 and Day 28; Scale bar: 50 μm). The semi‐quantitative score of IHC staining for Ac p53 K381 was in the graph, right. ^*^ *P* < .05 vs Day 0. n = 6 per group

In our present study, fenestrae in hepatic sinusoidal endothelium disappeared entirely on the 6th day (Figure 1A), along with the high expression of α‐SMA and vWF in CCl_4_‐induced rat models on the 28th day (Appendix Figure S1A‐C, E). Meanwhile, the serum ALT and AST levels increased, along with the augment of NOX2 protein expression in primary HSECs which were isolated from CCl_4_‐induced rat models (Appendix Figure S1D,E). These data indicated that CCl_4_ induced defenestration and capillarization in HSECs via oxidative damage. Interestingly, the senescence‐associated β‐galactosidase (SA‐β‐Gal)‐positive cells increased in primary HSECs; moreover, the Western blotting and immunofluorescence showed that a time‐dependent elevation of the progerin protein expression in HSECs of CCl_4_‐induced rat models (Figure 1B‐D). The data implied that progerin might be closely associated to premature senescence in CCl_4_‐induced defenestrated HSECs. However, the SIRT1 expression was down‐regulated, with the enhancement of the protein levels of Ac p53 K381 and total p53 in primary HSECs of CCl_4_‐induced rat models (Figure 1C); the immunofluorescence and the immunohistochemical staining showed less expression of SIRT1 but much expression of Ac p53 K381 in vWF‐positive hepatic sinusoidal endothelium (Figure 1E,F). Hence, these results confirmed that in the process of CCl_4_‐induced defenestration in HSECs and liver fibrosis, oxidative damage triggered progerin‐associated premature senescence, with the decrease of SIRT1‐mediated deacetylation.

### 3.2 Overexpression of SIRT1 inhibits progerin‐associated premature senescence to alleviate CCl_4_‐induced HSECs defenestration and liver fibrogenesis

**FIGURE 2:**
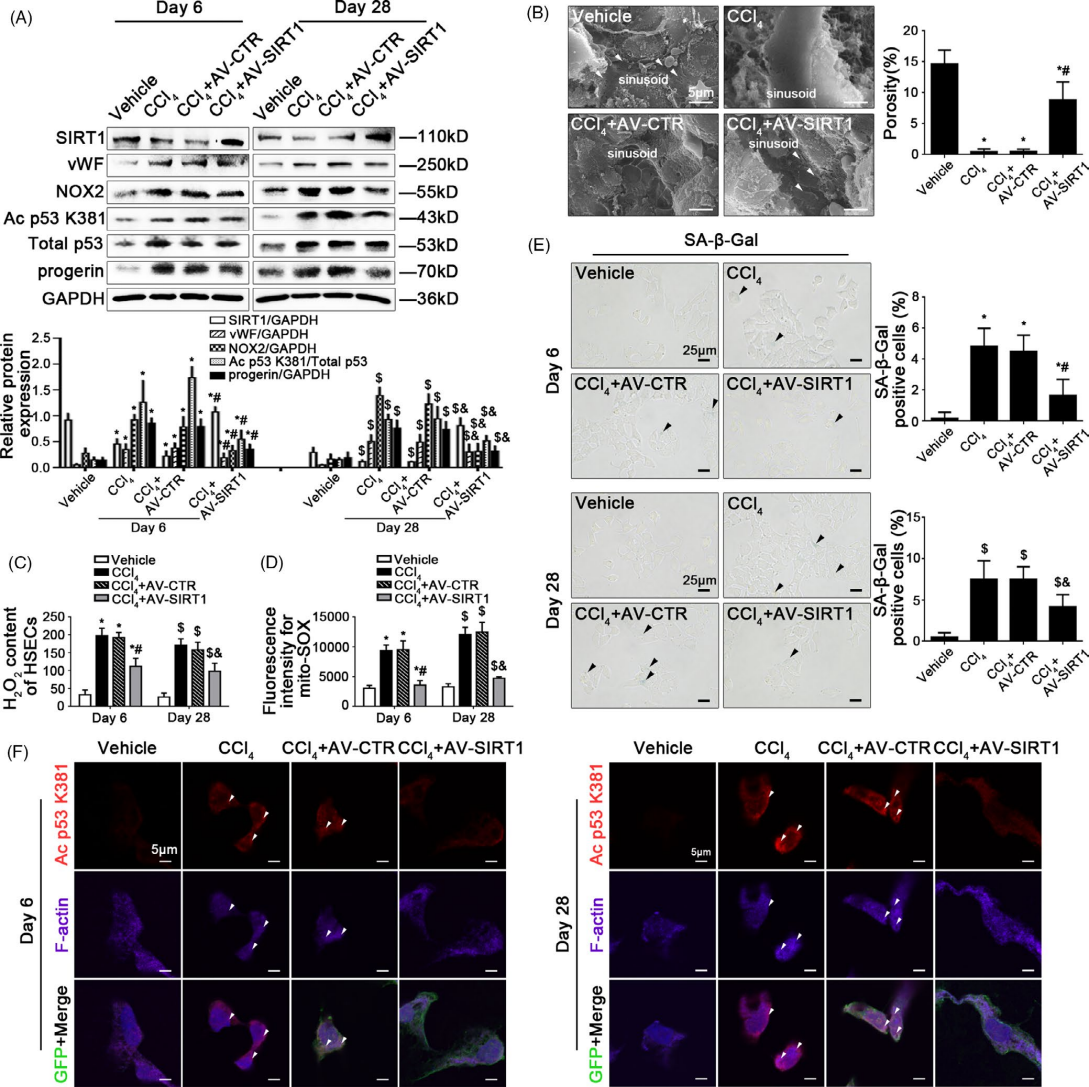
Overexpression of SIRT1 relieves progerin‐associated premature senescence to attenuate CCl_4_‐induced defenestration in HSECs. (A) Representative immunoblots of SIRT1, vWF, NOX2, Ac p53 K381, total p53 and progerin of primary HSECs, isolated from CCl_4_‐induced rat models on Day 6 and Day 28. The relative protein expression of SIRT1, vWF, NOX2 and progerin, as well as the ratio of Ac p53 K381 and total p53 protein levels were quantified in the graph, down. ^*^ *P* < .05 vs the vehicle group on Day 6; ^#^ *P* < .05 vs the CCl_4_ group and the CCl_4_ + AV‐CTR group on Day 6; ^$^ *P* < .05 vs the vehicle group on Day 28; ^&^ *P* < .05 vs the CCl_4_ group and the CCl_4_ + AV‐CTR group on Day 28. (B) Magnification of SEM of hepatic sinusoidal endothelium in CCl_4_‐induced rat models on Day 6, revealing fenestrae structures in hepatic sinusoidal endothelium (Scale bar: 5 μm). The white triangles indicated fenestrae in hepatic sinusoidal endothelium. The porosity of hepatic sinusoidal endothelium was quantified in the graph, right. ^*^ *P* < .05 vs the vehicle group; ^#^ *P* < .05 vs the CCl_4_ group and the CCl_4_ + AV‐CTR group. (C) The H_2_O_2_ content of primary HSECs, isolated from CCl_4_‐induced rat models on Day 6 and Day 28. ^*^ *P* < .05 vs the vehicle group on Day 6; ^#^ *P* < .05 vs the CCl_4_ group and the CCl_4_ + AV‐CTR group on Day 6; ^$^ *P* < .05 vs the vehicle group on Day 28; ^&^ *P* < .05 vs the CCl_4_ group and the CCl_4_ + AV‐CTR group on Day 28. (D) Fluorescence intensity for mito‐SOX of primary HSECs, isolated from CCl_4_‐induced rat models on Day 6 and Day 28, measuring with flow cytometry. ^*^ *P* < .05 vs the vehicle group on Day 6; ^#^ *P* < .05 vs the CCl_4_ group and the CCl_4_ + AV‐CTR group on Day 6; ^$^ *P* < .05 vs the vehicle group on Day 28; ^&^ *P* < .05 vs the CCl_4_ group and the CCl_4_ + AV‐CTR group on Day 28. (E) The SA‐β‐Gal activity on primary HSECs, isolated from CCl_4_‐induced rat models on Day 6 and Day 28, was observed by SA‐β‐Gal staining (Scale bar: 25 μm). The black triangles indicated the SA‐β‐Gal‐positive cells. ^*^ *P* < .05 vs the vehicle group on Day 6; ^#^ *P* < .05 vs the CCl_4_ group and the CCl_4_ + AV‐CTR group on Day 6; ^$^ *P* < .05 vs the vehicle group on Day 28; ^&^ *P* < .05 vs the CCl_4_ group and the CCl_4_ + AV‐CTR group on Day 28. (F) The immunocytochemical co‐localization of Ac p53 K381 (red) with F‐actin (purple) of primary HSECs, isolated from CCl_4_‐induced rat models on Day 6 and Day 28, visualized by confocal microscopy (Scale bar: 5 μm). Adenovirus vectors were showed by GFP (green). Nuclear was showed by DAPI (blue). n = 6 per group

To evaluate the role of SIRT1‐mediated deacetylation in premature senescence and HSECs defenestration in vivo, the SIRT1 adenoviral vector was in advance transferred to CCl_4_‐induced liver fibrogenesis rat models to ubiquitously overexpress SIRT1. There were about 70% of HSECs infected after injection. The H&E staining showed that the SIRT1 adenoviral vector attenuated CCl_4_‐induced liver acute injury and fibrogenesis on the 6th and the 28th day (Appendix Figure S2A). The vWF expression and the data of SEM demonstrated that overexpressing SIRT1 with adenoviral vector could maintain fenestrae and reduce capillarization in hepatic sinusoidal endothelium (Figure 2A,B; Appendix Figure S2B). Meanwhile, compared to the vehicle group, the NOX2 protein level, the H_2_O_2_ content and mito‐ROS increased in primary HSECs of the CCl_4_ group and the CCl_4_ + AV‐CTR group; these effects were reduced by the SIRT1 adenoviral vector (Figure 2A,C and D), indicated inhibition of oxidative damage via overexpressing SIRT1. Furthermore, compared to the vehicle group, the protein levels of Ac p53 K381, total p53, and progerin, as well as the SA‐β‐Gal‐positive cells were elevated in primary HSECs of the CCl_4_ group and the CCl_4_ + AV‐CTR group on the 6th and the 28th day, which were inhibited by overexpressing SIRT1 (Figure 2A,E; Appendix Figure S2C). Besides, the immunofluorescence showed that compared with the vehicle group, the co‐localization of Ac p53 K381 with F‐actin highly expressed in the nuclear envelope of HSECs in the CCl_4_ group and the CCl_4_ + AV‐CTR group on the 6th and 28th day; in contrast, in the CCl_4_ + AV‐SIRT1 group on the 6th and 28th day, less Ac p53 K381 co‐localized with F‐actin; meanwhile, F‐actin distributed uniformly around the cell membrane of HSECs (Figure 2F), implied that overexpressing SIRT1 with adenoviral vector attenuated progerin‐associated premature senescence and F‐actin remodelling to maintain fenestrae in HSECs.

Taken together, these results demonstrated that activating SIRT1‐mediated deacetylation relieved progerin‐associated premature senescence and maintained cytoskeleton to attenuate CCl_4_‐induced defenestration in hepatic sinusoidal endothelium and liver fibrogenesis.

### 3.3 Progerin‐associated premature senescence emerges in the process of defenestration in HSECs in vitro

**FIGURE 3:**
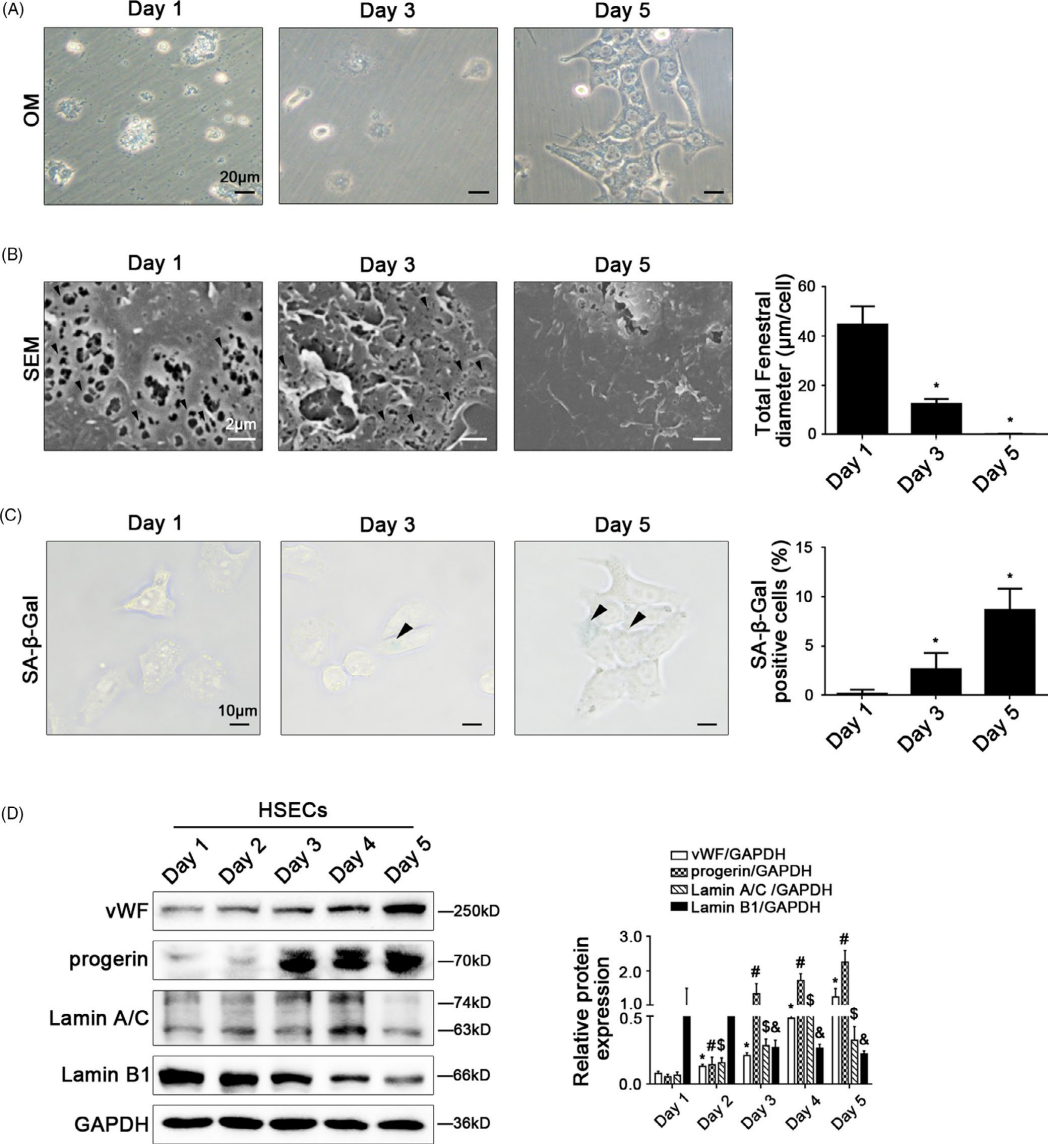
Progerin‐associated premature senescence emerges in the process of defenestration in HSECs in vitro. Freshly primary HSECs, isolated from normal rats, were cultured without growth factors in vitro for 5 days. (A) The morphology of HSECs on Day 1, Day 3 and Day 5 observed by the microscope (Scale bar: 20 μm). (B) Magnification of SEM of HSECs on Day 1, Day 3 and Day5, revealing fenestrae structures in HSECs (Scale bar: 2 μm). The black triangles indicated fenestrae in HSECs. The total fenestral diameter was quantified in the graph, right. ^*^ *P* < .05 vs Day 1. (C) The SA‐β‐Gal activity of HSECs on Day 1, Day 3 and Day 5 observed by SA‐β‐Gal staining (Scale bar: 10 μm). The black triangles indicated the SA‐β‐Gal‐positive cells. The SA‐β‐Gal‐positive cells were quantified in the graph, right. ^*^ *P* < .05 vs Day 1. (D) Representative immunoblots of vWF, progerin, Lamin A/C and Lamin B1 of primary HSECs. The relative protein expression was quantified in the graph, right. ^*^ *P* < .05 vs vWF protein level on Day 1; ^#^ *P* < .05 vs progerin protein level on Day 1; ^$^ *P* < .05 vs Lamin A/C protein level on Day 1; ^&^ *P* < .05 vs Lamin B1 protein level on Day 1

In vitro, the fenestrae in freshly primary HSECs, which were isolated from normal rats and were cultured without growth factors for 5 days (Figure 3A), shrank rapidly from the 1st day till the 3rd day and disappeared completely on the 5th day (Figure 3B). Interestingly, the SA‐β‐Gal‐positive cells increased gradually with time (Figure 3C), along with the elevated protein levels of vWF, progerin and Lamin A/C. In contrast, Lamin B1 protein expression was down‐regulated (Figure 3D). These results indicated that the defenestration in HSECs was probably related to progerin‐associated premature senescence.

### 3.4 Oxidative stress aggravates progerin‐associated premature senescence to facilitate defenestration in HSECs via acetylation of p53

**FIGURE 4:**
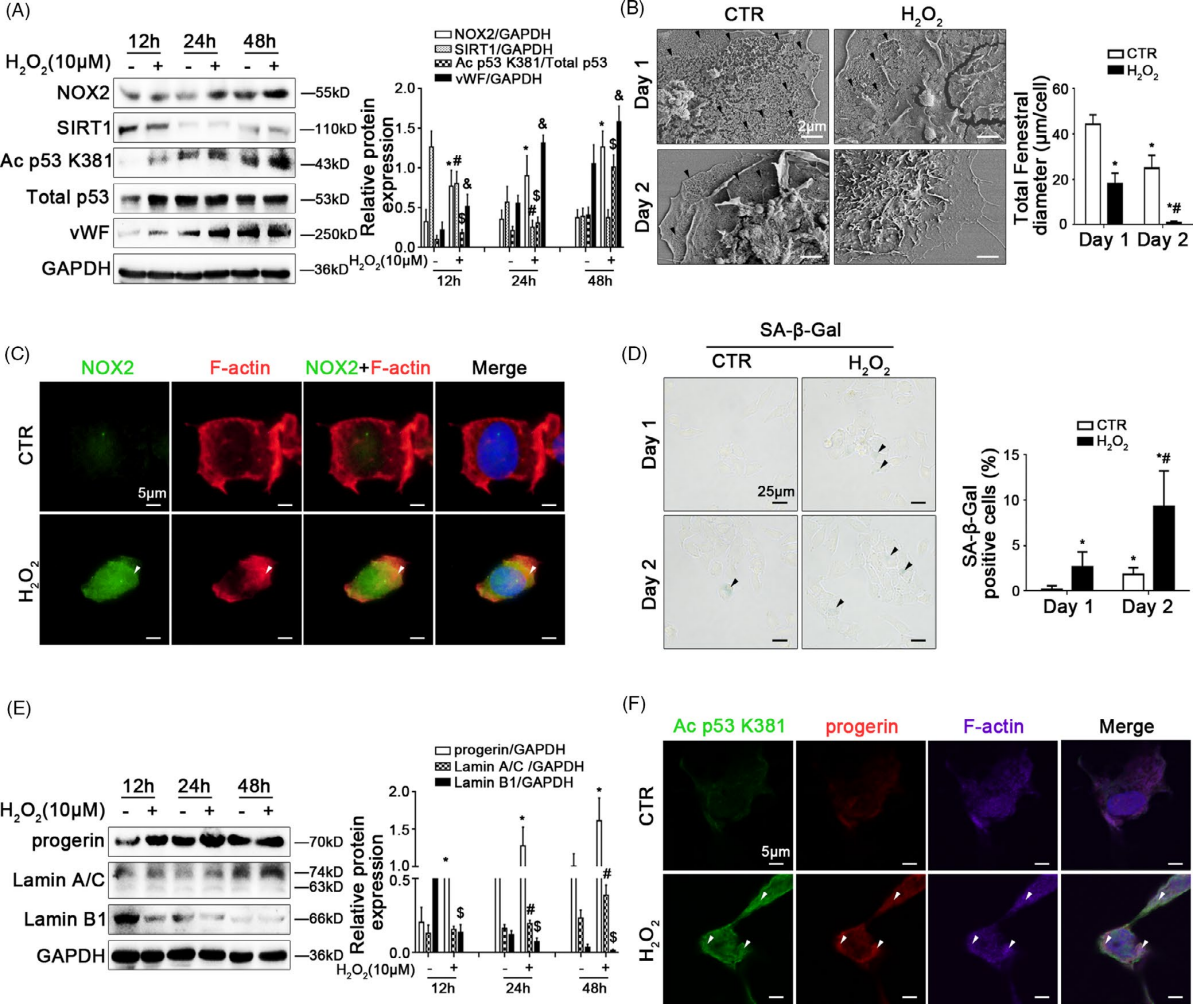
Oxidative stress inhibits SIRT1‐mediated deacetylation and aggravates progerin‐associated premature senescence to facilitate defenestration in HSECs. Freshly primary HSECs, isolated from normal rats and cultured in vitro, were treated with H_2_O_2_ (10 μM) from 12 hours to 48 hours. (A) Representative immunoblots of NOX2, SIRT1, Ac p53 K381, total p53 and vWF of HSECs in 12 hours, 24 hours and 48 hours. The relative protein expression of NOX2, SIRT1 and vWF, as well as the ratio of Ac p53 K381 and total p53 protein levels were quantified in the graph, right. ^*^ *P* < .05 vs NOX2 protein level in the concurrent control group; ^#^ *P* < .05 vs SIRT1 protein level in the concurrent control group; ^$^ *P* < .05 vs the ratio of Ac p53 K381 and total p53 protein levels in the concurrent control group; ^&^ *P* < .05 vs vWF protein level in the concurrent control group. (B) Magnification of SEM of HSECs in the CTR group and the H_2_O_2_ (10 μM) group on Day 1 and Day 2, revealing the fenestrae structures (Scale bar: 2 μm). The black triangles indicated fenestrae in HSECs. The total fenestral diameter was quantified in the graph, right. ^*^ *P* < .05 vs the CTR group on Day 1; ^#^ *P* < .05 vs the CTR group on Day 2. (C) The immunocytochemical co‐localization of NOX2 (green) with F‐actin (red) in primary HSECs on Day 2 visualized by confocal microscopy (Scale bar: 5 μm). Nuclear was showed by DAPI (blue). (D) The SA‐β‐Gal activity in primary HSECs on Day 1 and Day 2 was observed by SA‐β‐Gal staining (Scale bar: 25 μm). The black triangles indicated the SA‐β‐Gal‐positive cells. The SA‐β‐Gal‐positive cells were quantified in the graph, right. ^*^ *P* < .05 vs the CTR group on Day 1; ^#^ *P* < .05 vs the CTR group on Day 2. (E) Representative immunoblots of progerin, Lamin A/C and Lamin B1 of HSECs in 12 hours, 24 hours and 48 hours. The relative protein expression was quantified in the graph, right. ^*^ *P* < .05 vs progerin protein level in the concurrent control group; ^#^ *P* < .05 vs Lamin A/C protein level in the concurrent control group; ^$^ *P* < .05 vs Lamin B1 protein level in the concurrent control group. (F) The immunocytochemical co‐localization of Ac p53 K381 (green) with progerin (red) and F‐actin (purple) of primary HSECs on Day 2 visualized by confocal microscopy (Scale bar: 5 μm). Nuclear was showed by DAPI (blue)

Freshly primary HSECs, isolated from normal rats, were stimulated with H_2_O_2_ at the different doses (0, 1.25, 2.5, 5, 10 μM) for 24 hours or at the dose (10 μM) from 12 hours to 48 hours. There was a time‐dependent and a concentration‐dependent up‐regulation of NOX2, Ac p53 K381 and total p53 expression, with the decrease of SIRT1 expression, in H_2_O_2_‐treated primary HSECs (Figure 4A; Appendix Figure S3A). As expected, the data of SEM showed that the fenestrae in H_2_O_2_‐treated HSECs disappeared on the 2nd day, in advance of the control group (Figure 4B); meanwhile, the flow cytometry and immunocytochemistry showed that CD31, which labelled continuous HSECs, was highly expressed in H_2_O_2_‐treated primary HSECs (Appendix Figure S3B,C), with the augment of vWF protein level (Figure 4A). Furthermore, the immunofluorescence showed that compared with the control group, the co‐localization of NOX2 with F‐actin was highly expressed, accompany with the accumulation of F‐actin in the nuclear envelope of H_2_O_2_‐treated HSECs on the 2nd day (Figure 4C). These data suggested that H_2_O_2_‐induced oxidative damage might trigger the activation of acetylation of p53 and F‐actin remodelling to accelerate defenestration and capillarization in HSECs via NOX2.

Besides, the SA‐β‐Gal staining, and the protein levels of progerin, Lamin A/C and Lamin B1, showed that H_2_O_2_‐induced oxidative stress promoted progerin‐associated premature senescence in HSECs, with the decrease of Lamin B1 expression (Figure 4D,E). Compared to the control group, more progerin and Ac p53 K381 also were co‐localized with F‐actin in the nuclear envelope of H_2_O_2_‐treated HSECs on the 2nd day (Figure 4F). Hence, these results indicated that H_2_O_2_‐induced oxidative stress triggered progerin‐associated premature senescence through acetylation of p53 at lysine 381, and subsequently contributed to F‐actin remodelling to aggravate defenestration in HSECs.

In addition, primary HSECs were transfected with p53 siRNA, progerin siRNA or nontarget siRNA (called NC), and then administered with H_2_O_2_ (10 μM) for 2 days. We found that H_2_O_2_‐induced strengthened activity of SA‐β‐Gal was significantly reduced by silencing p53 with p53 siRNA (Appendix Figure S4A,B), suggested p53‐mediated premature senescence in H_2_O_2_‐treated HSECs. As expected, the data of SEM showed that silencing progerin with progerin siRNA attenuated H_2_O_2_‐induced HSECs defenestration on the 2nd day (Appendix Figure S4C and D), implied that inhibiting progerin‐associated premature senescence could maintain HSECs fenestrae.

In consequence, H_2_O_2_‐induced oxidative stress induced acetylation of p53 and progerin‐associated premature senescence, and then brought about abnormal cytoskeleton remodelling to aggravate defenestration in HSECs.

### 3.5 Inhibiting NOX2‐dependent oxidative stress reduces progerin‐associated premature senescence to maintain fenestrae in HSECs

**FIGURE 5:**
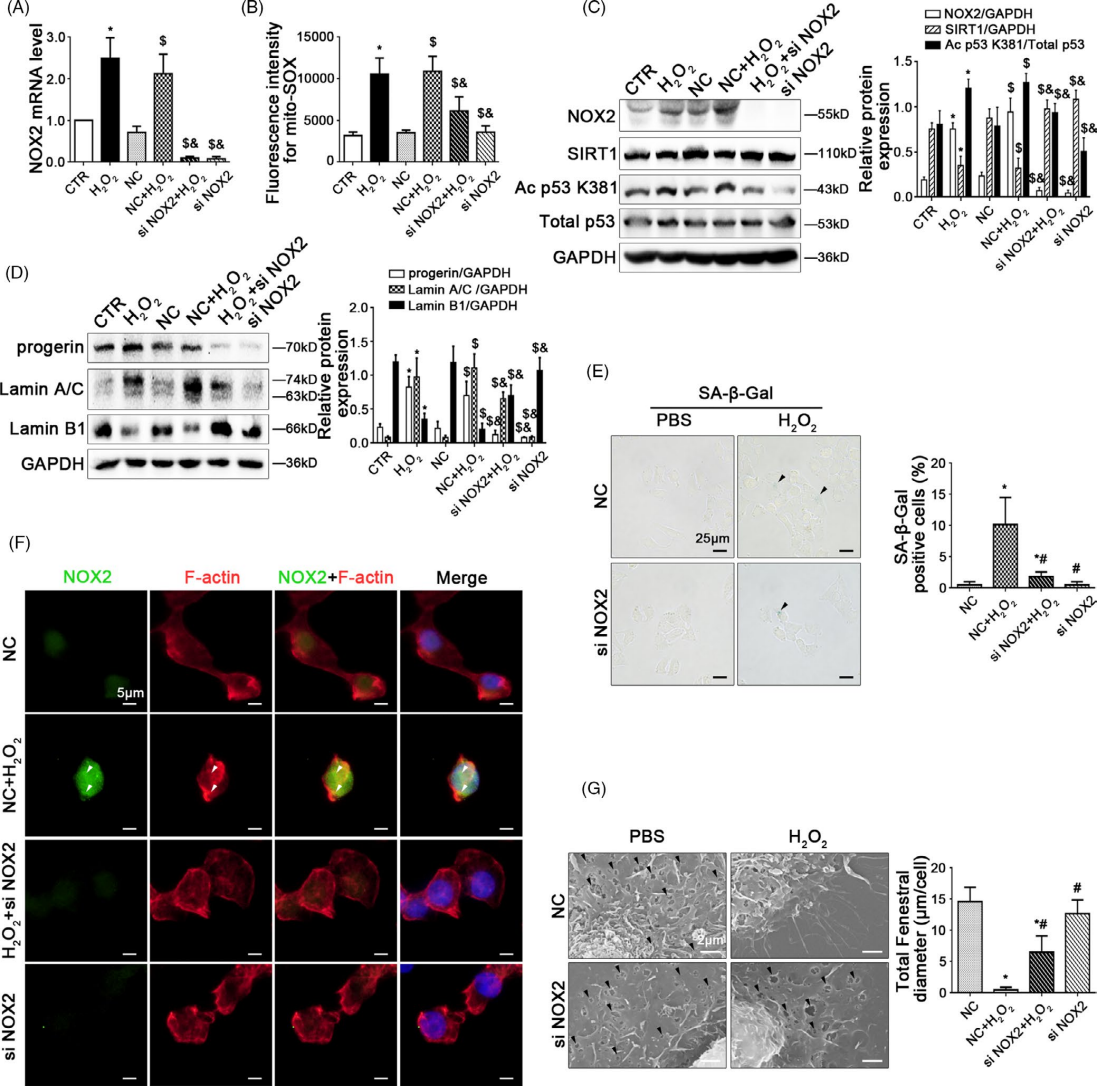
Inhibiting NOX2‐dependent oxidative stress reduces progerin‐associated premature senescence to maintain fenestrae in HSECs. Freshly primary HSECs, isolated from normal rats and cultured in vitro, were transfected with NOX2 siRNA or nontarget siRNA (called NC), and then administered with H_2_O_2_ (10 μM) for two days. (A) Real‐time PCR analysis of NOX2 mRNA level in HSECs on Day 2. ^*^ *P* < .05 vs the CTR group; ^$^ *P* < .05 vs the NC group; ^&^ *P* < .05 vs the NC + H_2_O_2_ group. (B) Fluorescence intensity for mito‐SOX of HSECs on Day 2, measuring with flow cytometry. ^*^ *P* < .05 vs the CTR group; ^$^ *P* < .05 vs the NC group; ^&^ *P* < .05 vs the NC + H_2_O_2_ group. (C) Representative immunoblots of NOX2, SIRT1, Ac p53 K381, and total p53 of HSECs on Day 2. The relative protein expression of NOX2 and SIRT1, as well as the ratio of Ac p53 K381 and total p53 protein levels, was quantified in the graph, right. ^*^ *P* < .05 vs the CTR group; ^$^ *P* < .05 vs the NC group; ^&^ *P* < .05 vs the NC + H_2_O_2_ group. (D) Representative immunoblots of progerin, Lamin A/C and Lamin B1 of HSECs on Day 2. The relative protein expression was quantified in the graph, right. ^*^ *P* < .05 vs the CTR group; ^$^ *P* < .05 vs the NC group; ^&^ *P* < .05 vs the NC + H_2_O_2_ group. (E) The SA‐β‐Gal activity in HSECs on Day 2 in the four groups (NC, NC + H_2_O_2_, H_2_O_2_ + si NOX2, si NOX2), was observed by the SA‐β‐Gal staining (Scale bar: 25 μm). The black triangles indicated the SA‐β‐Gal‐positive cells. The SA‐β‐Gal‐positive cells were quantified in the graph, right. ^*^ *P* < .05 vs the NC group; ^#^ *P* < .05 vs the NC + H_2_O_2_ group. (F) The immunocytochemical co‐localization of NOX2 (green) with F‐actin (red) in primary HSECs on Day 2, visualized by confocal microscopy (Scale bar: 5 μm). Nuclear were showed by DAPI (blue). (G) Magnification of SEM of HSECs in the four groups (NC, NC + H_2_O_2_, H_2_O_2_ + si NOX2, si NOX2), revealing fenestrae structures in HSECs (Scale bar: 2 μm). The black triangles indicated fenestrae in HSECs. The total fenestral diameter was quantified in the graph, right. ^*^ *P* < .05 vs the NC group; ^#^ *P* < .05 vs the NC + H_2_O_2_ group

To further delineate the molecular mechanism of oxidative stress‐induced premature senescence and defenestration in HSECs, primary HSECs, isolated from normal rats and cultured in vitro, were transfected with NOX2 siRNA or nontarget siRNA (called NC), and then administered with H_2_O_2_ (10 μM) for 2 days. The transfection efficiency achieved 75%. As we expected, compared to the control group and the NC group, H_2_O_2_ caused the increase of mito‐ROS, as well as the NOX2 mRNA and its high protein expression, as a result of NOX2‐dependent oxidative stress, along with the decrease of SIRT1 expression but the increase of Ac p53 K381 expression; in contrast, these effects remarkably were inhibited by knockdown of NOX2 with siRNA (Figure 5A‐C). Moreover, the protein levels of progerin and Lamin A/C, as well as the activity of SA‐β‐Gal were elevated in HSECs, along with the decrease of Lamin B1 expression; on the contrary, knockdown of NOX2 down‐regulated the protein levels of progerin and Lamin A/C, but up‐regulated the Lamin B1 level (Figure 5D and E), implied that inhibiting NOX2‐dependent oxidative damage attenuated progerin‐associated premature senescence. Additionally, the immunofluorescence and the SEM showed that accumulation of F‐actin in the nuclear envelope of H_2_O_2_‐treated HSECs and its defenestration, were triggered by oxidative stress, which were rescued by knockdown of NOX2 (Figure 5F and G). In short, inhibition of NOX2‐dependent oxidative stress alleviates progerin‐associated premature senescence to maintain fenestrae in HSECs.

### 3.6 SIRT1‐mediated deacetylation relieves NOX2‐dependent oxidative stress to maintain fenestrae in HSECs via attenuating progerin‐associated premature senescence

**FIGURE 6:**
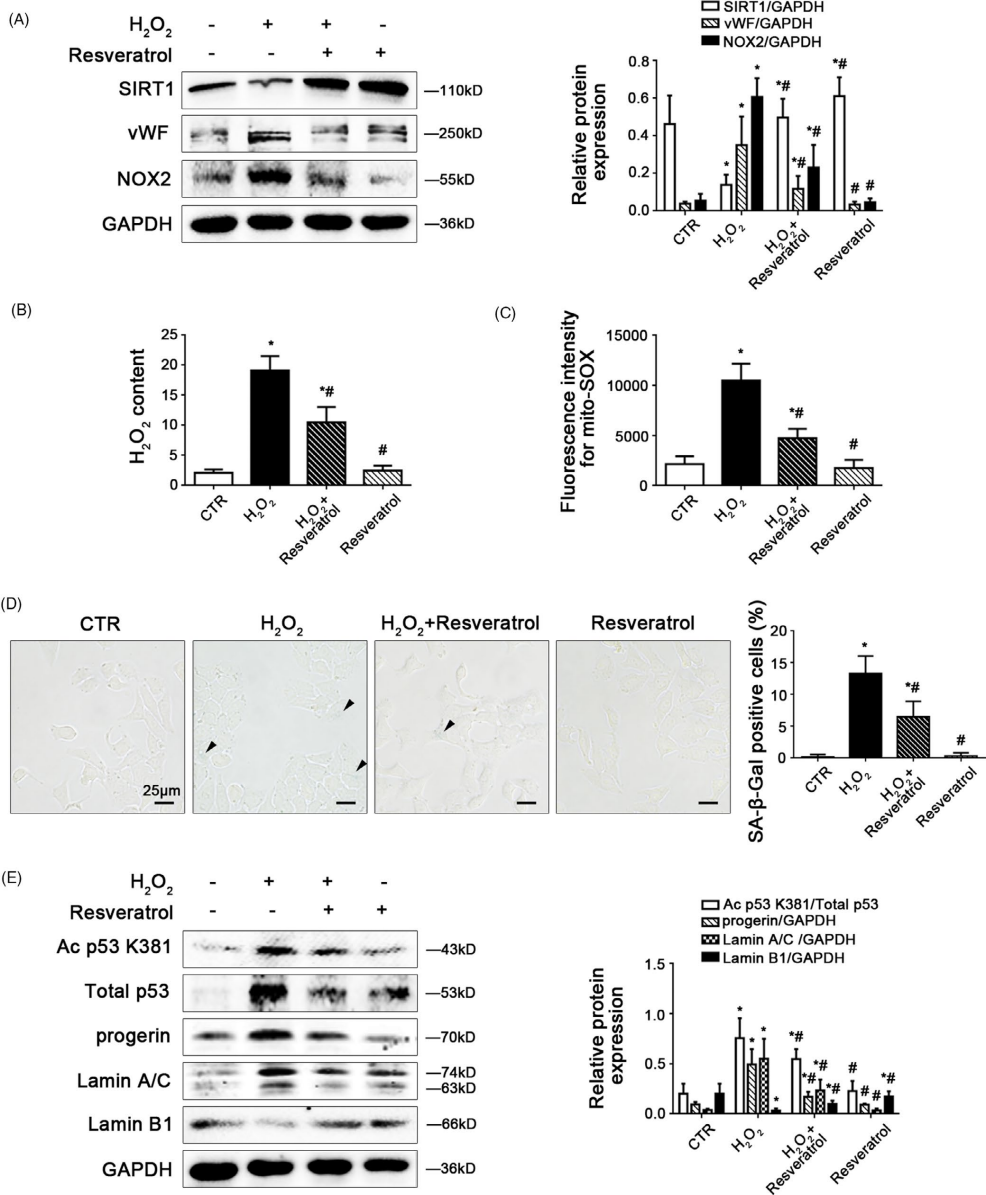
Activating SIRT1 with resveratrol reduces NOX2‐dependent oxidative stress and relieves progerin‐associated premature senescence. Freshly primary HSECs, isolated from normal rats and cultured in vitro, were treated with H_2_O_2_ (10 μM) and were simultaneously administered with resveratrol (a specific chemical activator of SIRT1, 1 μM) for two days. (A) Representative immunoblots of SIRT1, vWF and NOX2 of primary HSECs on Day 2 in four groups (CTR, H_2_O_2_, H_2_O_2_ + Resveratrol, Resveratrol). The relative protein expression was quantified in the graph, right. ^*^ *P* < .05 vs the CTR group; ^#^ *P* < .05 vs the H_2_O_2_ group. (B) The H_2_O_2_ content of primary HSECs on Day 2 in four groups (CTR, H_2_O_2_, H_2_O_2_ + Resveratrol, Resveratrol). ^*^ *P* < .05 vs the CTR group; ^#^ *P* < .05 vs the H_2_O_2_ group. (C) Fluorescence intensity for mito‐SOX of primary HSECs on Day 2 in four groups (CTR, H_2_O_2_, H_2_O_2_ + Resveratrol, Resveratrol), measuring with flow cytometry. ^*^ *P* < .05 vs the CTR group; ^#^ *P* < .05 vs the H_2_O_2_ group. (D) The SA‐β‐Gal activity in HSECs on Day 2 in the four groups (CTR, H_2_O_2_, H_2_O_2_ + Resveratrol, Resveratrol) was observed by SA‐β‐Gal staining (Scale bar: 25 μm). The black triangles indicated the SA‐β‐Gal‐positive cells. The SA‐β‐Gal‐positive cells were quantified in the graph, right. ^*^ *P* < .05 vs the CTR group; ^#^ *P* < .05 vs the H_2_O_2_ group. (E) Representative immunoblots of Ac p53 K381, total p53, progerin, Lamin A/C and Lamin B1 of primary HSECs on Day 2 in four groups (CTR, H_2_O_2_, H_2_O_2_ + Resveratrol, Resveratrol). The ratio of Ac p53 K381 and total p53 protein levels, as well as the relative protein expression of progerin, Lamin A/C and Lamin B1 were quantified in the graph, right. ^*^ *P* < .05 vs the CTR group; ^#^ *P* < .05 vs the H_2_O_2_ group

Firstly, to reveal the role of SIRT1 in oxidative stress‐triggered premature senescence in HSECs, primary HSECs, isolated from normal rats, were treated with H_2_O_2_ (10 μM) and were simultaneously administered with resveratrol (a specific chemical activator of SIRT1, 1 μM), or selisistat (a potent chemical inhibitor of SIRT1, 1 μM) for 2 days. The activation of SIRT1 was inhibited by H_2_O_2_, but was recovered by resveratrol (Figure 6A). Compared with the control group, the protein expression of vWF and NOX2, the H_2_O_2_ content, and mito‐ROS increased in the H_2_O_2_ group; in contrast, these effects were inhibited by resveratrol (Figure 6A‐C). Moreover, the activity of SA‐β‐Gal, the protein levels of Ac p53 K381, total p53, progerin, Lamin A/C were enhanced by H_2_O_2_, along with the decrease of Lamin B1; on the contrary, the effects were relieved by resveratrol (Figure 6D,E). The data indicated that activating SIRT1 with resveratrol could attenuate NOX2‐dependent oxidative stress and progerin‐associated premature senescence.

Besides, H_2_O_2_ down‐regulated the SIRT1 protein expression and enhanced the vWF protein level, and this effect was aggravated by selisistat, which implied that blocking the activity of SIRT1 might directly promote defenestration and capillarization in HSECs. However, selisistat did not alter the NOX2 protein expression, suggested that selisistat did not influence NOX2‐dependent oxidative stress (Appendix Figure S5A). As expected, the activity of SA‐β‐Gal, the protein levels of Ac p53 K381, total p53, progerin, Lamin A/C, and Lamin B1 showed that inhibiting SIRT1 with selisistat could directly mediate acetylation of p53 to exacerbate progerin‐associated premature senescence (Appendix Figure S5B,C).

**FIGURE 7:**
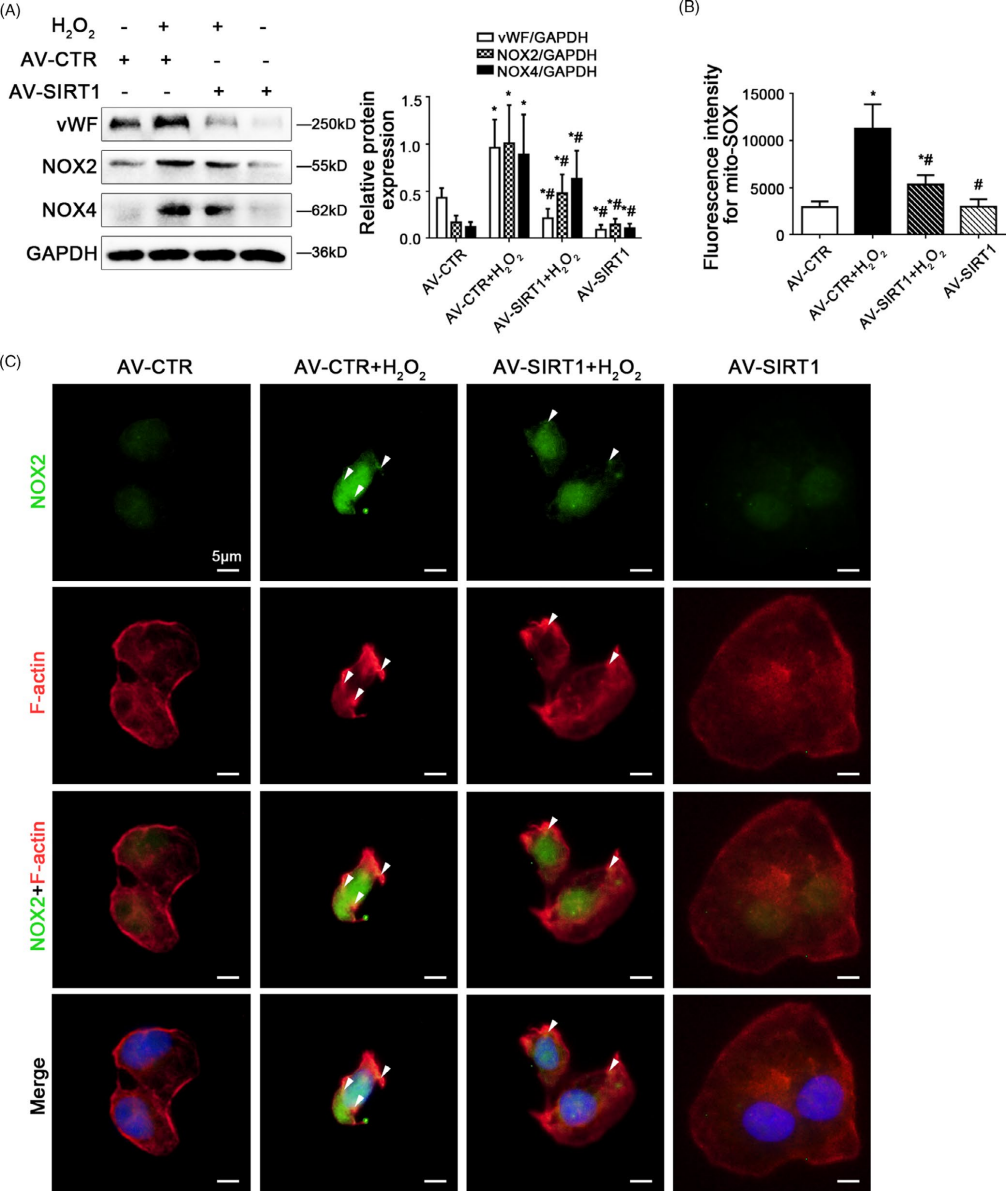
Overexpressing SIRT1 with adenovirus vector reduces F‐actin remodelling through inhibiting NOX2‐dependent oxidative stress. Freshly primary HSECs, isolated from normal rats and cultured in vitro, were transfected with the SIRT1 adenovirus vector to overexpress SIRT1 (called AV‐SIRT1) or nontarget adenovirus vector (called AV‐CTR), and then treated with H_2_O_2_ (10 μM) for two days. (A) Representative immunoblots of vWF, NOX2 and NOX4 of primary HSECs on Day 2 in four groups (AV‐CTR, AV‐CTR + H_2_O_2_, AV‐SIRT1 + H_2_O_2_, AV‐SIRT1). The relative protein expression was quantified in the graph, right. ^*^ *P* < .05 vs the AV‐CTR group; ^#^ *P* < .05 vs the AV‐CTR + H_2_O_2_ group. (B) Fluorescence intensity for mito‐SOX of primary HSECs on Day 2, measuring with flow cytometry. ^*^ *P* < .05 vs the AV‐CTR group; ^#^ *P* < .05 vs the AV‐CTR + H_2_O_2_ group. (C) The immunocytochemical co‐localization of NOX2 (green) with F‐actin (red) of primary HSECs on Day 2, visualized by confocal microscopy (Scale bar: 5 μm). Nuclear was showed by DAPI (blue)

Further, to characterize the specific mechanism about fenestrae in HSECs responding to SIRT1‐mediated deacetylation in depth, primary HSECs were transfected with the SIRT1 adenovirus vector to overexpress SIRT1 (namely AV‐SIRT1) or nontarget adenovirus vector (called AV‐CTR), and then stimulated with H_2_O_2_ (10 μM) for 2 days. The protein levels of vWF, NOX2, and NOX4, as well as mito‐ROS were enhanced by H_2_O_2_, which were decreased by overexpression of SIRT1 with adenovirus vector (Figure 7A and B), indicated that SIRT1 relieved H_2_O_2_‐induced NOX2‐dependent oxidative stress and mitochondrial dysfunction; meanwhile, compared with the control group, H_2_O_2_ caused more co‐localization of NOX2 with F‐actin in the nuclear envelope on the 2nd day; whereas these effects were suppressed by SIRT1 adenovirus vector (Figure 7C), implying that SIRT1 reduced F‐actin remodelling through inhibiting NOX2‐dependent oxidation.

**FIGURE 8:**
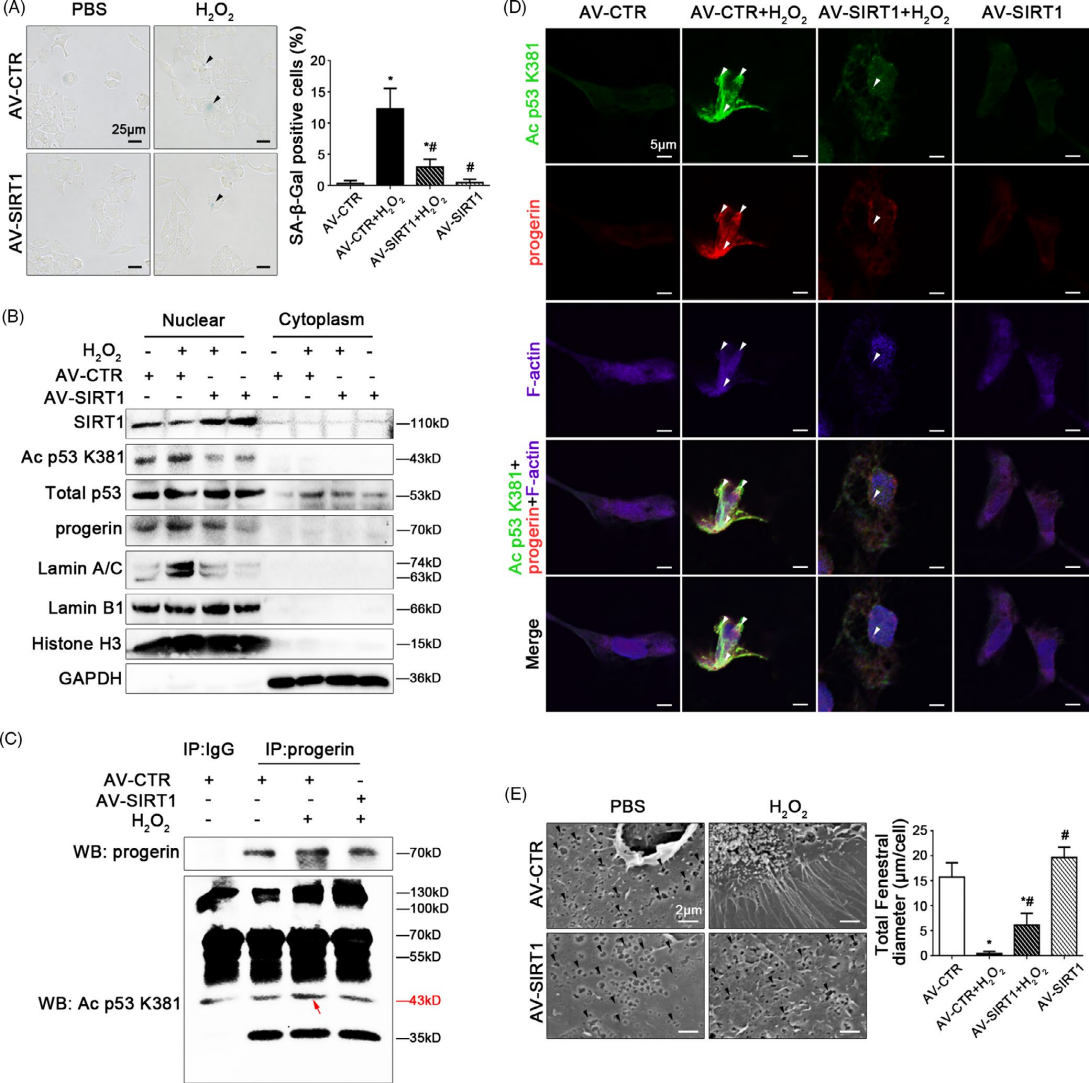
SIRT1‐mediated deacetylation maintains fenestrae in HSECs via inhibiting progerin‐associated premature senescence. Freshly primary HSECs, isolated from normal rats and cultured in vitro, were transfected with the SIRT1 adenovirus vector to overexpress SIRT1 (called AV‐SIRT1) or nontarget adenovirus vector (called AV‐CTR), and then treated with H_2_O_2_ (10 μM) for two days. (A) The SA‐β‐Gal activity in HSECs on Day 2 in the four groups (AV‐CTR, AV‐CTR + H_2_O_2_, H_2_O_2_ + AV‐SIRT1, AV‐SIRT1) was observed by SA‐β‐Gal staining (Scale bar: 25 μm). The black triangles indicated the SA‐β‐Gal‐positive cells. The SA‐β‐Gal‐positive cells were quantified in the graph, right. ^*^ *P* < .05 vs the AV‐CTR group; ^#^ *P* < .05 vs the AV‐CTR + H_2_O_2_ group. (B) Primary HSECs were extracted nuclear and cytoplasmic protein and were detected their protein levels. Representative immunoblots of SIRT1, Ac p53 K381, total p53, progerin, Lamin A/C and Lamin B1 in nuclei and cytoplasm of HSECs in four groups (AV‐CTR, AV‐CTR + H_2_O_2_, AV‐SIRT1 + H_2_O_2_, AV‐SIRT1). (C) Interaction of progerin with Ac p53 K381 was detected by the co‐IP assay. Progerin of HSECs was individually immunoprecipitated, as well as Ac p53 K381 and progerin subjected to immunoblotting analysis as indicated. (D) The immunocytochemical co‐localization of Ac p53 K381 (green) with progerin (red) and F‐actin (purple) in primary HSECs on Day 2 in four groups (AV‐CTR, AV‐CTR + H_2_O_2_, AV‐SIRT1 + H_2_O_2_, AV‐SIRT1), visualized by confocal microscopy (Scale bar: 5 μm). Nuclear was showed by DAPI (blue). (E) Magnification of SEM of HSECs in the four groups (AV‐CTR, AV‐CTR + H_2_O_2_, AV‐SIRT1 + H_2_O_2_, AV‐SIRT1), revealing fenestrae structures in HSECs (Scale bar: 2 μm). The black triangles indicated fenestrae in HSECs. The total fenestral diameter was quantified in the graph, right. ^*^ *P* < .05 vs the AV‐CTR group; ^#^ *P* < .05 vs the AV‐CTR + H_2_O_2_ group

Furthermore, the SA‐β‐Gal staining showed that overexpressing SIRT1 with the adenovirus vector reduced H_2_O_2_‐induced senescence (Figure 8A). The protein levels of SIRT1, Ac p53 K381, total p53, progerin, Lamin A/C, and Lamin B1 in nuclei and cytoplasm showed that compared to the control group, the expression of Ac p53 K381, progerin, and Lamin A/C were strongly elevated, with the decrease of SIRT1 and Lamin B1 in nuclei of H_2_O_2_‐treated HSECs; on the contrary, these effects were recused by overexpression of SIRT1 with adenovirus vector (Figure 8B), indicated that overexpression of SIRT1 suppressed progerin‐associated premature senescence. The co‐immunoprecipitation (Co‐IP) assay revealed that the co‐precipitation of Ac p53 K381 with progerin was enhanced in nuclei of H_2_O_2_‐treated HSECs, whose interaction was disrupted by SIRT1 adenovirus vector (Figure 8C); meanwhile, the immunofluorescence showed more Ac p53 K381 and progerin co‐localized with F‐actin in the nuclear envelope of H_2_O_2_‐treated HSECs on the 2nd day; whereas less co‐localization of Ac p53 K381 and progerin with F‐actin were displayed in the AV‐SIRT1 group and the AV‐SIRT1 + H_2_O_2_ group (Figure 8D). These data demonstrated that overexpressing SIRT1 blocked the nuclei Ac p53 K381‐progerin interaction. In addition, the data of SEM revealed that overexpression of SIRT1 with adenovirus vector rescued H_2_O_2_‐induced defenestration in HSECs (Figure 8E).

Therefore, these results confirmed that SIRT1‐mediated deacetylation of p53 relieved NOX2‐dependent oxidative stress and inhibited progerin‐associated premature senescence to maintain fenestrae in HSECs.

## 4 DISCUSSION

Our present study discovers that NOX2‐dependent oxidative stress triggers progerin‐associated premature senescence via acetylation of p53, and subsequently aggravates cytoskeleton remodelling to promote defenestration in HSECs; both activating SIRT1 with resveratrol and overexpressing SIRT1 with adenovirus vector strongly activates deacetylation of p53, and then relieves progerin‐related premature senescence to maintain fenestrae in HSECs and reverse liver fibrogenesis.

There is mounting evidence that premature senescence, induced by harmful stimuli, is a permanently deteriorated process about cell cycle arrest in advance, which involves in the dysfunction of endothelial cells and its related diseases as diverse as diabetes, lipodystrophy, and atherosclerosis, to name a few.^15^, ^16^ In our previous findings, premature senescence emerges in liver tissue due to acute liver injury and liver fibrogenesis.^17^ In addition, in older animal models and premature ageing‐related disease cases, abnormal differentiation and dysfunction in HSECs are attributed to premature senescence.^2^, ^3^ In this study, we similarly found that premature senescence in HSECs occurred in the process of fenestrae disappearance in vitro; and oxidative stress enhanced premature senescence to aggravate defenestration in H_2_O_2_‐treated HSECs and in HSECs of CCl_4_‐induced fibrogenesis. Nevertheless, the underlying mechanism about how oxidative stress‐induced premature senescence triggers HSECs defenestration is still elusive.

Lamins and its associated protein, especially Lamin A and Lamin B1, forms an interface with the nuclear membrane and nuclear pore complexes,^18^ while alteration of lamins involves in cellular premature senescence and age‐associated diseases. Interestingly, some literature regarding the influence of lamins on premature senescence and liver diseases are controversial. For instance, the disruption or mutation of lamins led to hepatocytes' abnormal differentiation and premature ageing to induce steatohepatitis.^7^ Progerin, a mutant of Lamin A, is deemed a moderator for premature ageing and dysfunction in endothelial cells.^19^ The depletion of Lamin B contributes to chronic inflammation.^20^ Our intriguing discoveries revealed that premature senescence in HSECs, along with the abnormal accumulation of progerin and the decrease of Lamin B1, during H_2_O_2_‐treated or CCl_4_‐induced HSECs defenestration; whereas knockdown of progerin could reduce premature senescence to recover fenestrae in H_2_O_2_‐treated HSECs. These findings indicate that A‐type and B‐type lamins are required for HSECs fenestrae; meanwhile, oxidative stress‐induced premature senescence leads to defenestration in HSECs via targeting progerin.

However, how progerin‐associated premature senescence, induced by oxidative stress, aggravates defenestration in HSECs? Chronic oxidative stress plays a role in liver inflammatory and liver fibrosis. NADPH oxidases (NOXs), mediating reactive oxygen species (ROS) generation, involves in chronic oxidative damage, hepatocytes apoptosis and HSCs activation to promote liver fibrogenesis.^21^ Many accumulating evidence conforms that various isoforms of NOXs family express in different hepatic cells. For example, NOX1, NOX2 and NOX4 are expressed in HSECs.^22^ During chronic liver diseases, NOX1, NOX2 and NOX4 are highly expressed in HSECs.^23^ During liver fibrogenesis, the redox signalling of different NOX isoforms and its effects on phenotypes and function of HSECs are unclear. Recent reports indicated that the augment of NOX1 in HSECs reduced the bioavailability of NO to induce cellular inflammation and liver injury.^24^ We previously found that activation of NOX4 in HSECs of either CCl_4_‐ or BDL‐induced liver fibrosis rat models generated abundant ROS to promote HSECs defenestration and liver fibrosis.^5^, ^6^ NOX2 activation and its derived ROS are implicated in oxidative damage of vascular function in ageing‐related diseases ^25^; activating NOX2 accelerates liver fibrosis during ageing.^26^ It is reported that NOX2 contributed to cytoskeleton rearrangement and capillarization of HSECs and its loss of scavenging function.^27^, ^28^ Little is known about the exact mechanism of NOX2 in HSECs defenestration and its premature senescence. Our findings showed that H_2_O_2_ elevated the NOX2 protein level and mitochondrial dysfunction to trigger progerin‐associated premature senescence, leading to defenestration in HSECs; silencing NOX2 could rescue mitochondrial function and premature senescence, and subsequently maintain HSECs fenestrae. These data demonstrated that premature senescence and defenestration in HSECs, which were induced by oxidative damage, attributed to NOX2 activation.

Besides, emerging reports show that lamins play a critical role in nucleoskeleton and cytoskeleton; whereas, accumulation of progerin contributes to perturbations in actin organization,^19^ implying that progerin‐associated premature senescence modulates cytoskeleton to affect cell phenotype. Some novel studies report that lamins‐related ageing is regulated by transcription factors (such as p53, NF‐κB, etc).^29^, ^30^ In our present study, more co‐localization of NOX2 and F‐actin displayed in the nuclear envelope of H_2_O_2_‐treated HSECs, whose fenestrae disappeared; on the contrary, knockdown of NOX2, which reduced oxidative stress‐induced premature senescence, inhibited F‐actin remodelling to rescue defenestration in HSECs. In brief, NOX2‐dependent oxidative stress triggered progerin‐associated premature senescence to result in defenestration in HSECs via F‐actin remodelling.

SIRT1, a class Ⅲ histone deacetylase, serves as an important modulator of metabolism, cellular survival and lifespan.^10^ Some recent evidence reports that the activation of SIRT1 confers protective effects on hepatic senescence and HSCs activation to ameliorate liver fibrosis.^11^, ^12^ The previous investigations reveal that SIRT1 protects vascular endothelial cells against age‐related endothelial dysfunction, whereas little research explores the role of SIRT1 in the morphology and function of HSECs. In our present study, oxidative damage activated acetylation of p53 in H_2_O_2_‐treated HSECs; in parallel, the Ac p53 K381 protein expression highly expressed in nuclei and co‐localized with more progerin and F‐actin in the nuclear envelope of H_2_O_2_‐treated HSECs. However, activating SIRT1 with resveratrol or overexpression of SIRT1 with adenovirus vector inhibited NOX2‐dependent oxidative stress and relieved premature senescence via deacetylation of p53; whereas inhibiting SIRT1 aggravated progerin‐associated premature senescence. Furthermore, overexpression of SIRT1 inhibited abnormal accumulation of progerin and F‐actin remodelling, to attenuate H_2_O_2_‐treated and CCl_4_‐induced HSECs defenestration and liver fibrogenesis. It follows that activation of SIRT1 effectively protects against defenestration in HSECs through inhibiting progerin‐associated premature senescence.

Although this study provides new insights into the mechanisms of premature senescence in HSECs defenestration, there are still some limitations to our present study. The mechanism about the reduction of Lamin B1 in HSECs defenestration needs to lucubrate. Besides, how progerin‐associated premature senescence affects liver sinusoidal capillarization remains elusive.

In conclusion, NOX2‐dependent oxidative damage aggravates defenestration in HSECs due to progerin‐associated premature senescence; SIRT1‐mediated deacetylation of p53 maintains fenestrae in HSECs and attenuates liver fibrogenesis via inhibition of progerin‐associated premature senescence.

## 5 CONCLUSIONS

Taken together, our data confirm that NOX2‐dependent oxidative damage aggravates HSECs defenestration via progerin‐associated premature senescence; SIRT1‐mediated deacetylation of p53 maintains fenestrae and attenuates liver fibrogenesis through inhibiting oxidative stress‐induced premature senescence.

## CONFLICT OF INTERESTS

The authors declare that they have no conflict of interest.

## AUTHOR CONTRIBUTIONS

Xiaoying Luo designed the research, conceived ideas, performed experiments, wrote the manuscript and obtained funding. Yangqiu Bai, Xiaoke Jiang and Shuli He performed experiments and analysed data. Zhiyu Yang, Di Lu, Suofeng Sun, Peiru Wei, Yuan Liang, Cong Peng, Ruli Sheng, Yaru Wang, Shuangyin Han and Xiuling Li critically revised the manuscript. Bingyong Zhang designed the research, conceived ideas and directed the study. All authors edited and reviewed the final manuscript.

## Supporting information

Supplementary Material

## Data Availability

The data sets generated during and/or analysed during the current study are available from the corresponding author upon request.
