# Supplementary material for: Sirtuin 1 ameliorates defenestration in hepatic sinusoidal endothelial cells during liver fibrosis via inhibiting stress‐induced premature senescence
Source: Cell Prolif. 2021 Feb 1;54(3):e12991. doi: 10.1111/cpr.12991 (PMC7941223; doi:10.1111/cpr.12991)
Supplement: Supplementary file 1 — Supplementary Material [file CPR-54-e12991-s001.docx]

**Supplementary**

**Supplementary methods**

**An Ishak inflammation and fibrosis score for CCl_4_-induced liver histological grading and staging.**

The rat liver histological inflammation and fibrosis stage are assessed according to the reference grading system (Ishak score) (Nakaji et al., 2002), considering the score listed in Table 1 and Table 2.

**Table 1 Hepatic necroinflammation scores**

| **Score** | **Change** | | | |
| --- | --- | --- | --- | --- |
|  | **Periportal or periseptal interface hepatitis (piecemeal necrosis)** | **Confluent necrosis** | **Focal (spotty) lytic necrosis, apoptosis and focal inflammation** | **Portal inflammation** |
| 0 | Absent | Absent | Absent | None |
| 1 | Mild (focal, few portal areas) | Focal confluent necrosis | One focus or less per 10×objective | Mild, some or all portal areas |
| 2 | Mild/moderate (focal, most portal areas) | Zone 3 necrosis in some areas | Two to four foci per 10×objective | Moderate, some or all portal areas |
| 3 | Moderate (continuous around <50% of tracts or septa) | Zone 3 necrosis in most areas | Five to ten foci per 10×objective | Moderate/marked, all portal areas |
| 4 | Severe (continuous around >50% of tracts or septa) | Zone 3 necrosis +occasional portal-central (P-C) bridging | More than ten foci per 10×objective | Marked, all portal areas |
| 5 | - | Zone 3 necrosis +multiple P-C bridging | - | - |
| 6 | - | Panacinar or multiacinar necrosis | - | - |

**Table 2 Hepatic fibrosis scores**

| **Score** | **Change** |
| --- | --- |
| 0 | No fibrosis |
| 1 | Fibrous expansion of some portal areas, with or without short fibrous septa |
| 2 | Fibrous expansion of most portal areas, with or without short fibrous septa |
| 3 | Fibrous expansion of most portal areas, with occasional portal to portal (P-P) bridging |
| 4 | Fibrous expansion of portal areas with marked bridging (portal to portal (P-P) as well as portal to central (P-C)) |
| 5 | Marked bridging (P-P and/or P-C) with occasional nodules (incomplete cirrhosis) |
| 6 | Cirrhosis, probable or definite |

**Supplementary figure legends:**

**
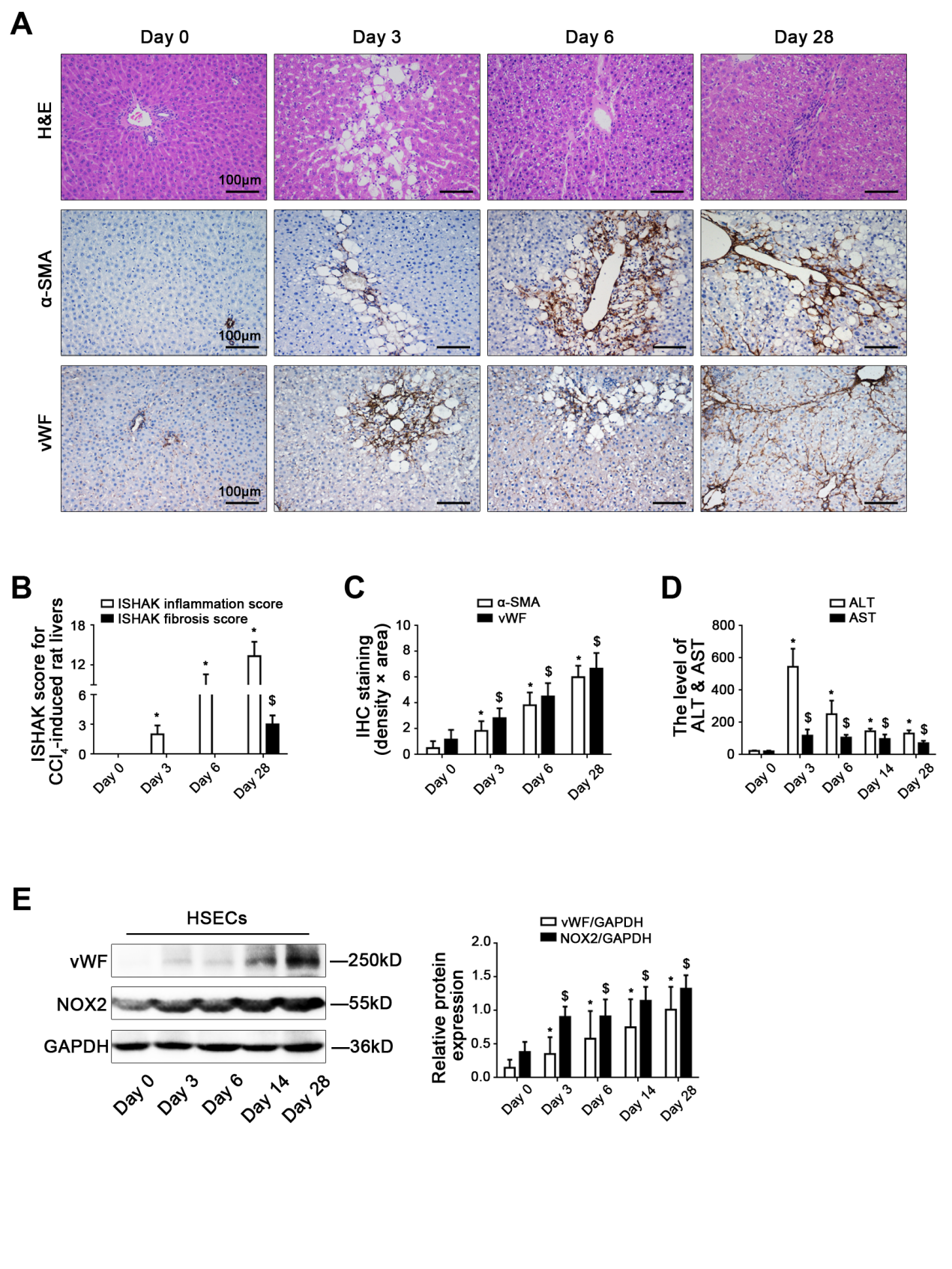
**

**Supplementary Figure 1 Oxidative stress increased in HSECs, along with liver sinusoidal endothelium capillarization during CCl_4_-induced liver fibrogenesis.**

(**A**) The H&E staining, and immumohistochemical (IHC) staining for α-SMA and vWF of liver biopsy specimens of CCl_4_-induced rat models (Day 0, Day 3, Day 6, and Day 28) (Scale bar: 100 μm). (**B**) The ISHAK score of liver biopsy specimens of CCl_4_-induced rat models. ^*^P<0.05 versus the ISHAK inflammation score on Day 0; ^$^P<0.05 versus the ISHAK fibrosis score on Day 0. (**C**) The area density of IHC staining for α-SMA and vWF. ^*^P<0.05 versus the area density of α-SMA in Day 0; ^$^P<0.05 versus the area density of vWF in Day 0. (**D**) The ALT and AST levels of CCl_4_-induced rat models (Day 0, Day 3, Day 6, Day 14, and Day 28). ^*^P<0.05 versus the ALT level on Day 0; ^$^P<0.05 versus the AST level on Day 0. (**E**) Representative immunoblots of vWF and NOX2 of primary HSECs isolated from the CCl_4_-induced rat models. (Day 0, Day 3, Day 6, Day 14, and Day 28). The relative protein expression was quantified in the graph, right. ^*^P<0.05 versus the vWF protein level on Day 0; ^$^P<0.05 versus the NOX2 protein level on Day 0. n=6 per group.


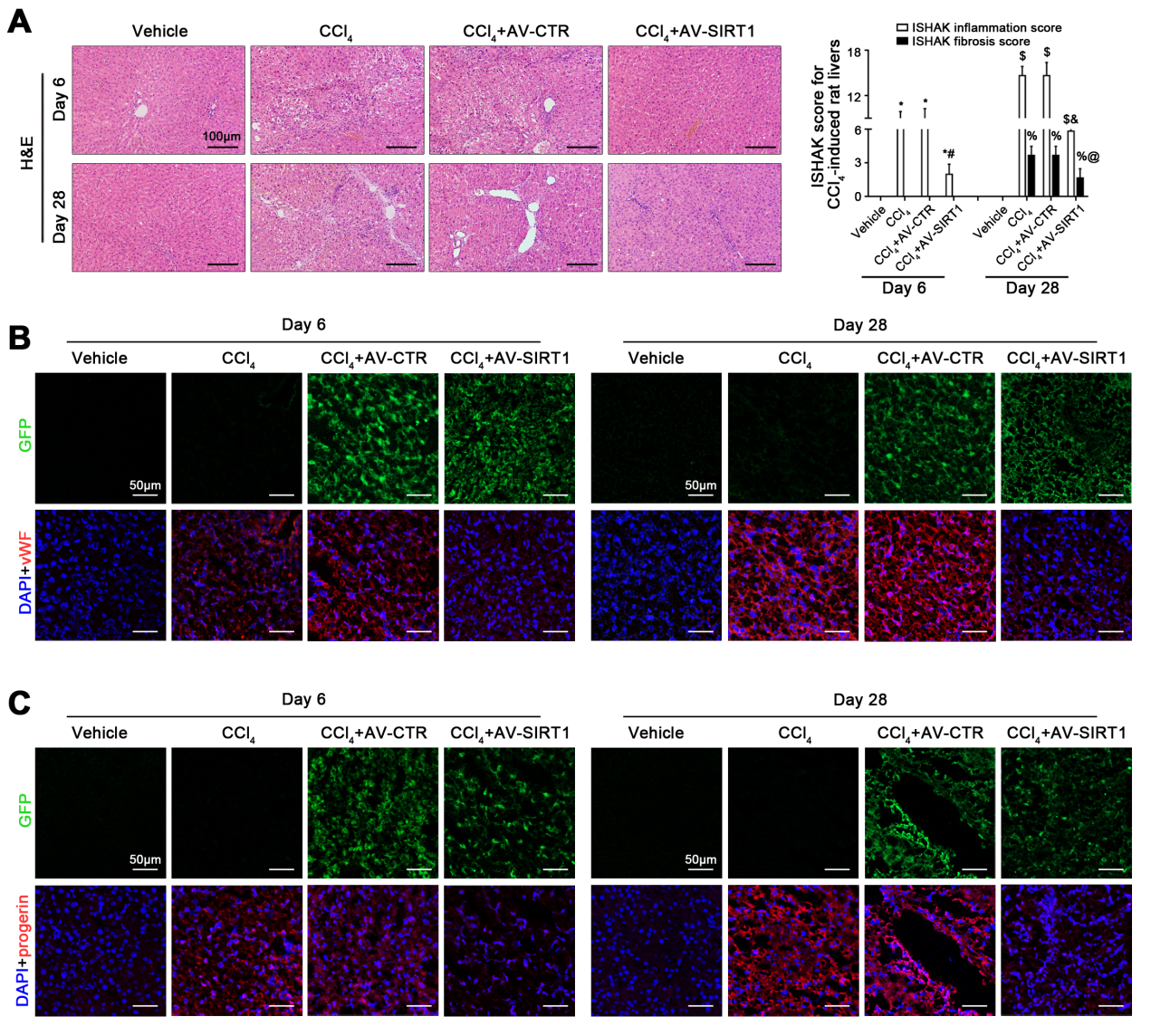


**Supplementary Figure 2 Overexpression of SIRT1 alleviates premature senescence in capillarized liver sinusoidal endothelium and CCl_4_-induced liver fibrogenesis.**

(**A**) The H&E staining of liver biopsy specimens of CCl_4_-induced rat models on Day 6 and Day 28 (Scale bar: 100 μm). The ISHAK score of liver biopsy specimens of CCl_4_-induced rat models on Day 6 and Day 28 was quantified in the graph, right. ^*^P<0.05 versus the ISHAK inflammation score of the vehicle group on Day 6; ^#^P<0.05 versus the ISHAK inflammation score of the CCl_4_ group and the CCl_4_+AV-CTR group on Day 6; ^$^P<0.05 versus the ISHAK inflammation score of the vehicle group on Day 28; ^&^P<0.05 versus the ISHAK inflammation score of the CCl_4_ group and the CCl_4_+AV-CTR group on Day 28; ^%^P<0.05 versus the ISHAK fibrosis score of the vehicle group on Day 28; ^@^P<0.05 versus the ISHAK fibrosis score of the CCl_4_ group and the CCl_4_+AV-CTR group on Day 28. (**B**) The immunofluorescent (IF) staining for vWF of liver biopsy specimens of CCl_4_-induced rat models on Day 6 and Day 28 (Scale bar: 50 μm). Adenovirus vectors were showed by GFP. Green represented the GFP expression, meanwhile red represented the vWF protein expression, visualized by confocal microscopy. Nuclear were showed by DAPI (blue). (**C**) The immunofluorescent staining for progerin of liver biopsy specimens of CCl_4_-induced rat models on Day 6 and Day 28 (Scale bar: 50 μm). Adenovirus vectors were showed by GFP. Green represented the GFP expression, meanwhile red represented the progerin protein expression, visualized by confocal microscopy. Nuclear were showed by DAPI (blue). n=6 per group.

**
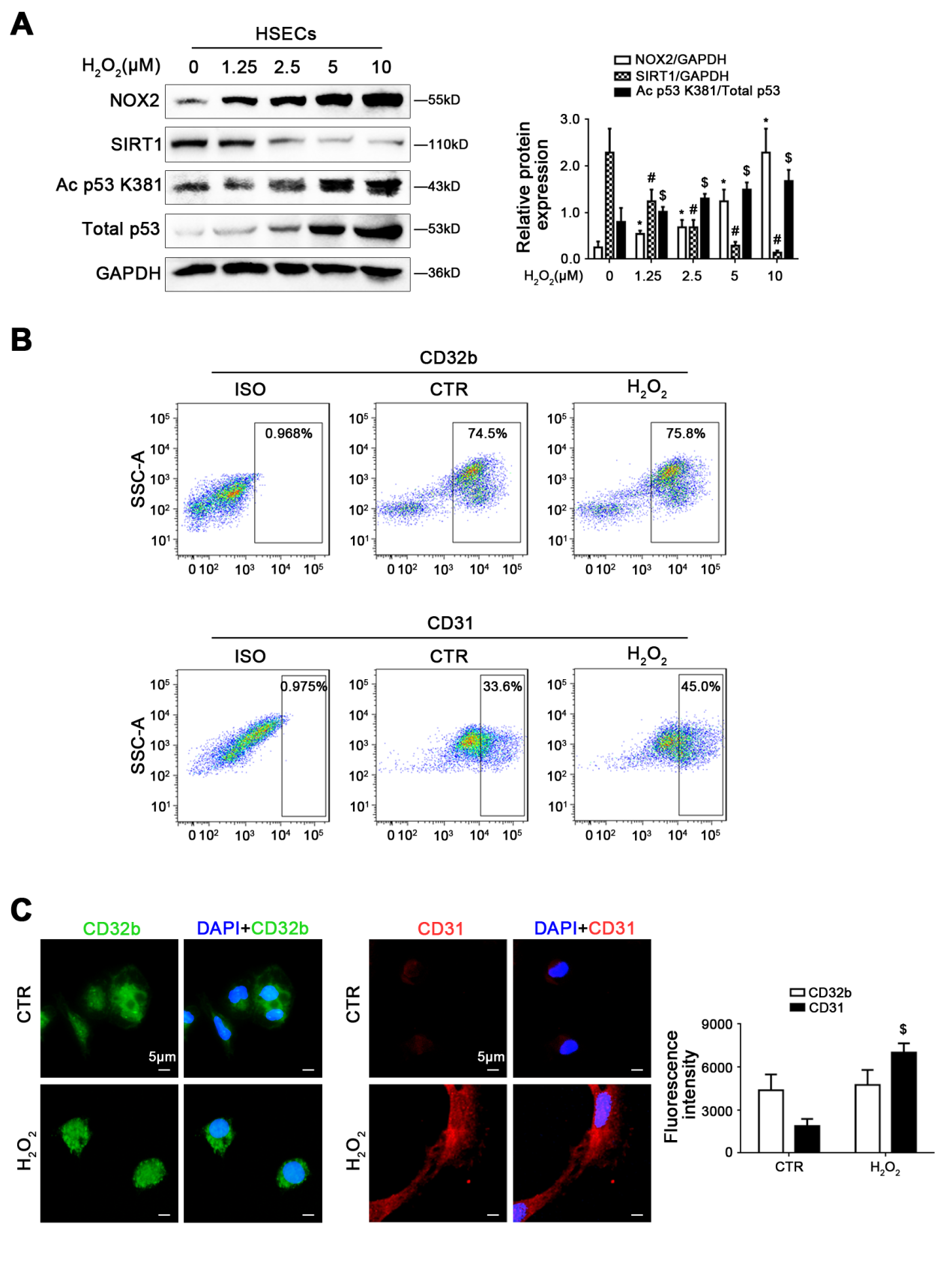
**

**Supplementary Figure 3 H_2_O_2_-induced oxidative stress inhibits acetylation of p53 to accelerate capillarization in HSECs.**

Freshly primary HSECs, isolated from normal rats, were cultured and stimulated with H_2_O_2_ at the different doses (0, 1.25, 2.5, 5, 10 μM) for 24 hours. (**A**) Representative immunoblots of NOX2, SIRT1, Ac p53 K381, and total p53 of HSECs. The relative protein expression of NOX2 and SIRT1, as well as the ratio of Ac p53 K381 and total p53 protein levels were quantified in the graph, right. ^*^P<0.05 versus NOX2 protein level of the 0 μM group; ^#^P<0.05 versus SIRT1 protein level of the 0 μM group; ^$^P<0.05 versus the ratio of Ac p53 K381 and total p53 protein levels of the 0 μM group. (**B**) The expression of CD32b and CD31 on HSECs measuring with flow cytometry. (**C**) The protein levels of CD32b (green) and CD31 (red) of HSECs detecting with immunocytochemistry (Scale bar: 5 μm). Nuclear were showed by DAPI (blue). Fluorescence intensity was quantified in the graph, right. ^*^P<0.05 versus CD32b protein level of the CTR group; ^$^P<0.05 versus CD31 protein level of the CTR group. CD32b, also called HSECs Fc-receptor (FcγIIb2), was a specific rat HSECs surface marker, while CD31 labeled continuous HSECs.


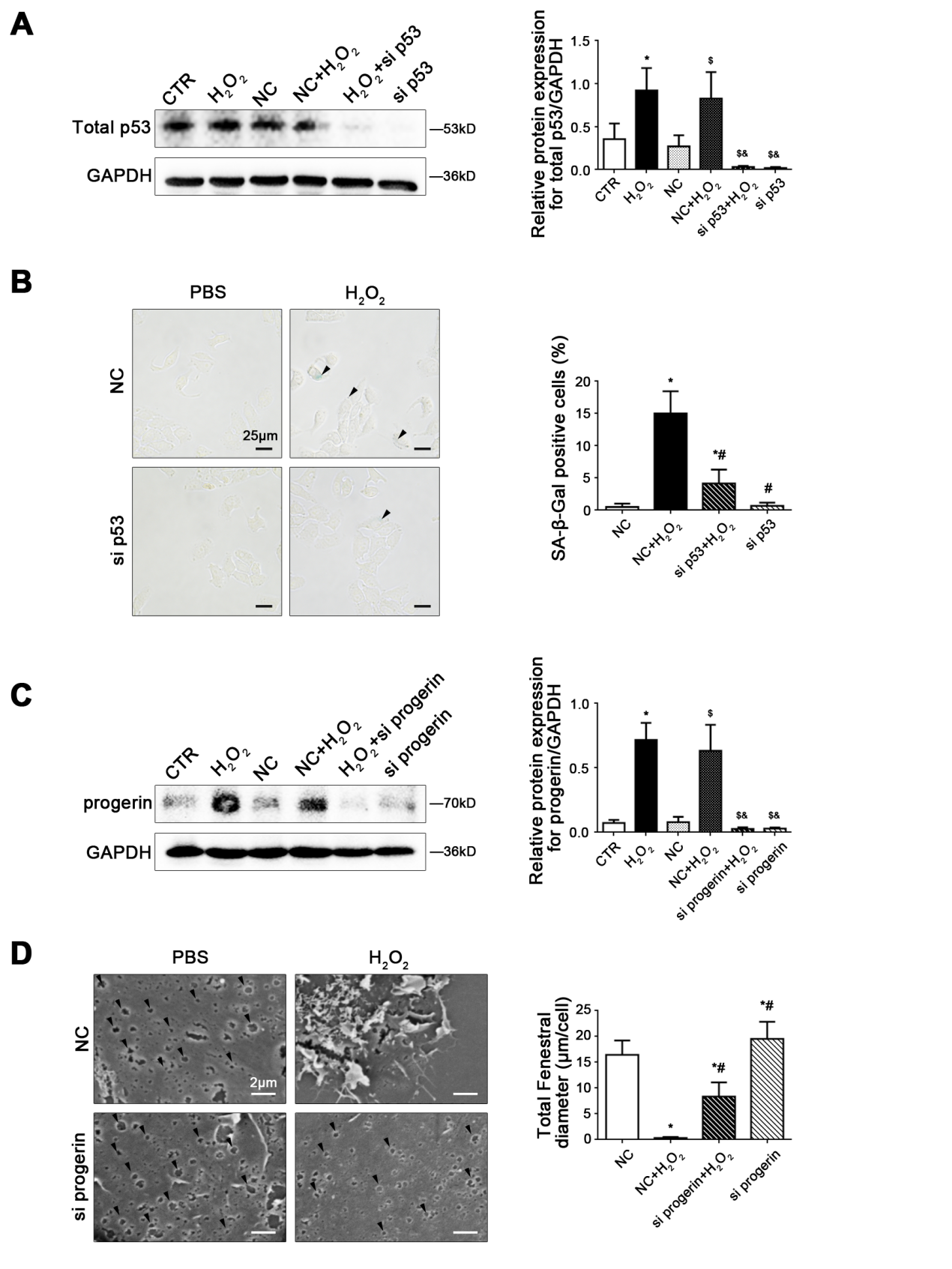


**Supplementary Figure 4 H_2_O_2_ induces progerin-associated premature senescence to promote defenestration in HSECs via p53.**

Freshly primary HSECs, isolated from normal rats and cultured *in vitro*, were transfected with p53 siRNA, progerin siRNA, or nontarget siRNA (called NC), and then administered with H_2_O_2_ (10 μM) for two days. (**A**) The representative immunoblot of p53. The relative protein expression was quantified in the graph, right. ^*^P<0.05 versus the CTR group; ^$^P<0.05 versus the NC group; ^&^P<0.05 versus the NC+H_2_O_2_ group. (**B**) The SA-β-Gal activity in HSECs of the four groups (NC, NC+H_2_O_2_, H_2_O_2_+si p53, si p53), was observed by the SA-β-Gal staining (Scale bar: 25 μm). The black triangles indicated the SA-β-Gal-positive cells. The SA-β-Gal-positive cells were quantified in the graph, right. ^*^P<0.05 versus the NC group; ^#^P<0.05 versus the NC+H_2_O_2_ group. (**C**) The representative immunoblot of progerin. The relative protein expression was quantified in the graph, right. ^*^P<0.05 versus the CTR group; ^$^P<0.05 versus the NC group; ^&^P<0.05 versus the NC+H_2_O_2_ group. (**D**) Magnification of scanning electron micrograph (SEM) of HSECs in the four groups (NC, NC+H_2_O_2_, H_2_O_2_+si progerin, si progerin), revealing fenestrae structures in HSECs (Scale bar: 2 μm). The black triangles indicated fenestrae in HSECs. The total fenestral diameter was quantified in the graph, right. ^*^P<0.05 versus the NC group; ^#^P<0.05 versus the NC+H_2_O_2_ group.


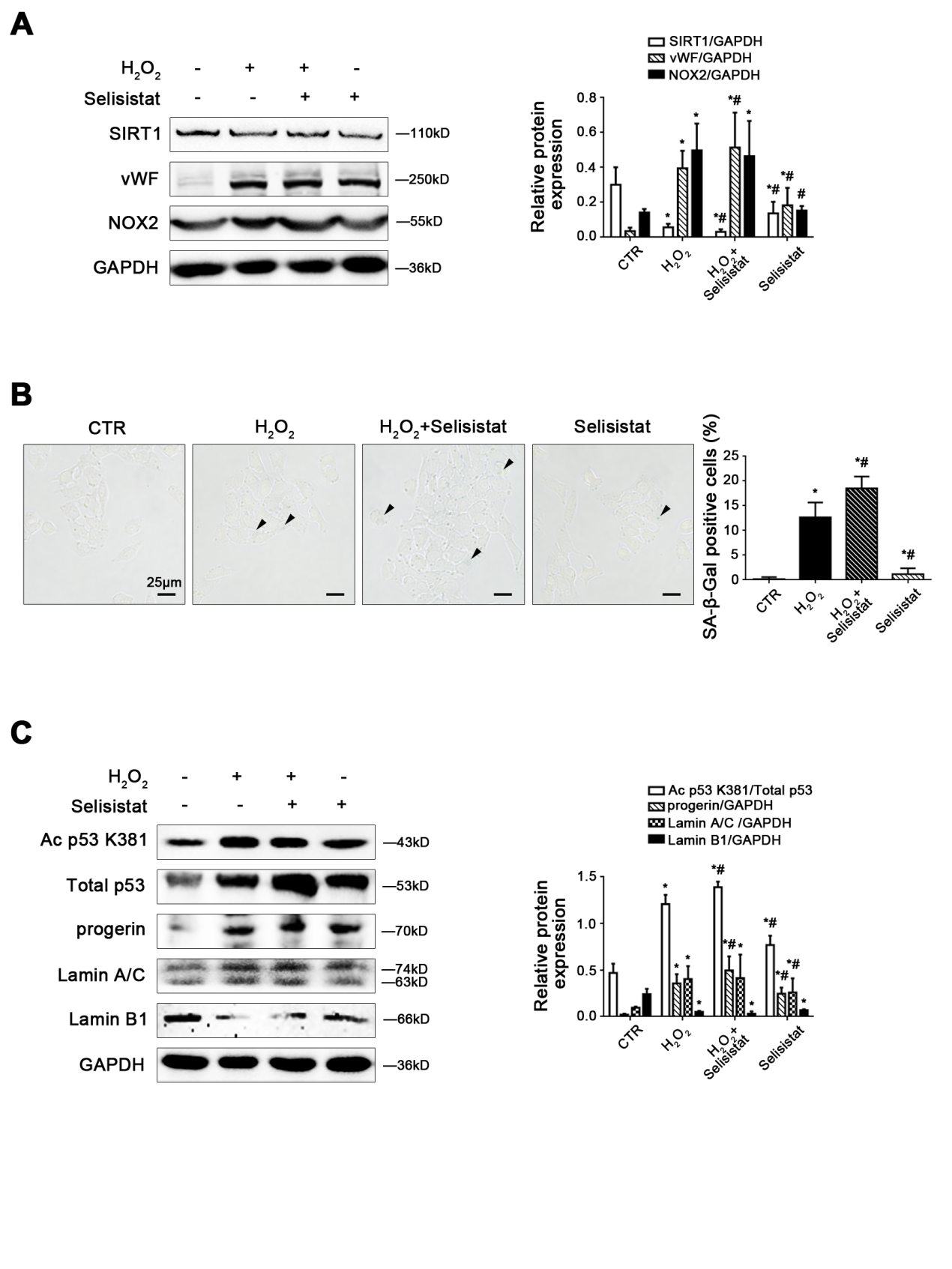


**Supplementary Figure 5 Blocking SIRT1 with selisistat exacerbates progerin-associated premature senescence.**

Freshly primary HSECs, isolated from normal rats and cultured *in vitro*, were treated with H_2_O_2_ (10 μM) and were simultaneously administered with selisistat (a chemical inhibitor of SIRT1, 1 μM) for two days. (**A**) Representative immunoblots of SIRT1, vWF, and NOX2 of primary HSECs on Day 2 in four groups (CTR, H_2_O_2_, H_2_O_2_+ Selisistat, Selisistat). The relative protein expression was quantified in the graph, right. ^*^P<0.05 versus the CTR group; ^#^P<0.05 versus the H_2_O_2_ group. (**B**) The SA-β-Gal activity in HSECs on Day 2 in the four groups (CTR, H_2_O_2_, H_2_O_2_+Selisistat, Selisistat), was observed by SA-β-Gal staining (Scale bar: 25 μm). The black triangles indicated the SA-β-Gal-positive cells. The SA-β-Gal-positive cells were quantified in the graph, right. ^*^P<0.05 versus the CTR group; ^#^P<0.05 versus the H_2_O_2_ group. (**C**) Representative immunoblots of Ac p53 K381, total p53, progerin, Lamin A/C, and Lamin B1 of primary HSECs on Day 2 in four groups (CTR, H_2_O_2_, H_2_O_2_+Selisistat, Selisistat). The ratio of Ac p53 K381 and total p53 protein levels, as well as the relative protein expression of progerin, Lamin A/C, and Lamin B1 were quantified in the graph, right. ^*^P<0.05 versus the CTR group; ^#^P<0.05 versus the H_2_O_2_ group.

**Reference**

Nakaji M, Hayashi Y, Ninomiya T, Yano Y, Yoon S, Seo Y, et al. Histological grading and staging in chronic hepatitis: its practical correlation. *Pathol Int*. 2002; 52: 683-690.
